# Development of Prognostic Features of Hepatocellular Carcinoma Based on Metabolic Gene Classification and Immune and Oxidative Stress Characteristic Analysis

**DOI:** 10.1155/2023/1847700

**Published:** 2023-02-18

**Authors:** Shimeng Cui, Minghao Zhang, Shanshan Bai, Yanfang Bi, Shan Cong, Shi Jin, Shuang Li, Hui He, Jian Zhang

**Affiliations:** ^1^Department of Hepatobiliary Surgery, The First Affiliated Hospital of Dalian Medical University, Dalian 116000, China; ^2^Department of Vascular Interventional, Affiliated Hongqi Hospital of Mudanjiang Medical College, Mudanjiang 157011, China; ^3^Department of Ultrasound, The First Affiliated Hospital of Dalian Medical University, Dalian 116000, China; ^4^Department of Nursing, The First Affiliated Hospital of Dalian Medical University, Dalian 116000, China; ^5^Department of Laparoscopic Surgery, The First Affiliated Hospital of Dalian Medical University, Dalian 116000, China; ^6^Bioinformatics R&D Department, Hangzhou Mugu Technology Co., Ltd., Hangzhou 310000, China; ^7^Department of Interventional Therapy, The First Affiliated Hospital of Dalian Medical University, Dalian 116000, China

## Abstract

**Background:**

The molecular classification of HCC premised on metabolic genes might give assistance for diagnosis, therapy, prognosis prediction, immune infiltration, and oxidative stress in addition to supplementing the limitations of the clinical staging system. This would help to better represent the deeper features of HCC.

**Methods:**

TCGA datasets combined with GSE14520 and HCCDB18 datasets were used to determine the metabolic subtype (MC) using ConsensusClusterPlus. ssGSEA method was used to calculate the IFN*γ* score, the oxidative stress pathway scores, and the score distribution of 22 distinct immune cells, and their differential expressions were assessed with the use of CIBERSORT. To generate a subtype classification feature index, LDA was utilized. Screening of the metabolic gene coexpression modules was done with the help of WGCNA.

**Results:**

Three MCs (MC1, MC2, and MC3) were identified and showed different prognoses (MC2-poor and MC1-better). Although MC2 had a high immune microenvironment infiltration, T cell exhaustion markers were expressed at a high level in MC2 in contrast with MC1. Most oxidative stress-related pathways are inhibited in the MC2 subtype and activated in the MC1 subtype. The immunophenotyping of pan-cancer showed that the C1 and C2 subtypes with poor prognosis accounted for significantly higher proportions of MC2 and MC3 subtypes than MC1, while the better prognostic C3 subtype accounted for significantly lower proportions of MC2 than MC1. As per the findings of the TIDE analysis, MC1 had a greater likelihood of benefiting from immunotherapeutic regimens. MC2 was found to have a greater sensitivity to traditional chemotherapy drugs. Finally, 7 potential gene markers indicate HCC prognosis.

**Conclusion:**

The difference (variation) in tumor microenvironment and oxidative stress among metabolic subtypes of HCC was compared from multiple angles and levels. A complete and thorough clarification of the molecular pathological properties of HCC, the exploration of reliable markers for diagnosis, the improvement of the cancer staging system, and the guiding of individualized treatment of HCC all gain benefit greatly from molecular classification associated with metabolism.

## 1. Introduction

Hepatocellular carcinoma (HCC) is ranked as the second major contributor to malignant tumors [1], and its etiology is closely related to viral infection and liver fibrosis [2]. Following surgery or ablation, around 70% of cases of HCC will recur within the first 5 years, and the 5-year survival rate is only approximately 30-40% [3]. The main factors leading to the dismal prognosis of HCC patients include an elevated degree of malignancy, easy recurrence, insensitivity to radiotherapy and chemotherapy, and proneness to vascular, lymphatic, and distant metastasis [4].

The tumor microenvironment (TME), especially the tumor immune microenvironment, performs an integral function in HCC. The TME comprises cancer cells, signaling molecules, fibroblasts, infiltrating immune cells, surrounding blood vessels, and the extracellular matrix [5]. Previous research has demonstrated that the TME may influence the gene expression of tumor tissues in a variety of ways, which could in turn influence the onset and progression of cancers [6]. For instance, tumor cells may modulate the TME via the negative control mechanism established by the immune system of the body. A wide variety of immunosuppressive states may be employed to counter the antitumor immunity that the body naturally has [7]. The degree of immunosuppression present in the TME is intimately linked to the individual differences in the effectiveness of tumor immunotherapy [5]. The dynamic network that constitutes the TME is primarily made up of stromal and immune cells that have infiltrated the tumor tissues. It is generally accepted that the mutual metabolic needs of immune and tumor cells are the root cause of immunosuppression in the surrounding environment [8], and inflammation performs a fundamental function in the development, invasion, and metastasis of HCC [9]. Although earlier research examined the tumor immune microenvironment (TIME) of HCC, further studies are needed to identify diagnostic markers for HCC.

Currently, clinicopathological staging is a standard method that is frequently utilized for determining the prognosis of individuals who have HCC. Nevertheless, HCC often exhibits clinical heterogeneity, which diminishes the efficacy of prognostic evaluations that are routinely performed. To provide high-risk groups with more clinically meaningful treatment strategies, prolong survival, and improve quality of life, the findings of the clinical staging prediction need to be optimized by the development of an innovative prognostic prediction model. As a result of the advent of gene chips and high-throughput sequencing technologies, as well as the large amounts of data included in the GEO and the TCGA databases, the systematic and thorough investigation of genes associated with tumors and the modulatory mechanisms that control them based on bioinformatic approaches has become an integral aspect of the present tumor genomics group. In the past ten years, screening for genetic alterations at the genome level has seen extensive use of gene sequencing and bioinformatic analysis, elucidating part of the molecular mechanism of the onset and advancement of HCC and guiding the determination of differentially expressed genes (DEGs) that participate in the progression of HCC. Functional pathway research provides more options for HCC therapy [10]. Recently, the identification of genetic markers for the prognosis of HCC has become a hot spot in many studies [11, 12]. However, because the properties of tumor markers are dependent on the tumor burden, their significance in the early diagnosis of tumors is limited.

The high proliferation and persistent inflammation of HCC cells are related to oxidative stress. In HCC, HBV genome-encoded X protein (HBx) is associated with increased ROS in mitochondria [13]. Increased levels of oxidative DNA damage, 8-oxyoguanine (8-oxoG), were observed in human hepatocellular carcinoma cells infected with HCV and in the liver of transgenic mice expressing HCV core protein in vitro [14]. Site-specific epigenetic alterations in HCC cells include methylation of the E-cadherin promoter caused by H_2_O_2_ treatment and methylation of the cytokine signaling suppressor SOCS3 caused by HBV-induced mitochondrial ROS accumulation [15]. In general, increased ROS levels are one of the causes of HCC proliferative.

As a crucial aspect of malignancies, metabolic disorders are an important factor to consider [16], since they all influence various HCC biological behaviors, development, and transfer recurrence [17, 18]. Carcinogenic factors, on the one hand, disturb the body's delicate metabolic equilibrium and lead to the development of metabolic recombinant cell carcinoma; in addition, the metabolic system after recombination is mediated by various biological behaviors, participating in the proliferative, invasive, and metastatic abilities of cancer cells [19, 20]. Recently, researchers have investigated the development of the molecular pathological properties of HCC [21]. These studies have summarized many abnormal metabolic genes related to HCC prognosis from different cells, animals, or HCC [22, 23]. In summary, the goal of HCC metabolism research is to fully understand the molecular pathological characteristics of HCC, explore reliable markers of HCC diagnosis and transfer recurrence prediction, improve the hepatoma staging system, guide individualization, and improve the treatment of HCC [24].

Based on this purpose, we divided HCC into different metabolic subtypes and multidimensional differences between different metabolic subtypes. Different MCs were analyzed, and different response patterns were observed with immunotherapy. At the same time, the correlation between the immunological examination points and the distinct metabolic molecular types and the variation in molecular mutations were subjected to a comparison. The final screen was selected with the potential prognostic marker associated with the metabolic feature index. In summary, we have created numerous subtypes of an immunological festival index and developed a molecular categorization model premised on metabolic properties, thus supplementing the lack of clinical installment systems. Our findings will serve as a base of research directions as well as a theoretical foundation for the evaluation of prognosis and the tailored therapy of HCC patients.

## 2. Materials and Methods

### 2.1. Sources of Expression Profile Information and Genes Involved in Metabolism

We retrieved TCGA-HCC RNA-seq data as well as clinical survival and characteristic data by utilizing the TCGA GDC API. We downloaded datasets with survival time including the GSE14520 from the Gene Expression Omnibus (GEO) database and the HCCDB18 from the HCCDB database (http://lifeome.net/database/hccdb/home.html). The date of the data retrieval was the 7th of April 2021. We downloaded the metabolism-related genes corresponding to the keywords carbohydrates, oxidations, glycogen, glycogenolysis, glycolysis, pyruvate, citric acid, and fatty acid from the KEGG and Reactome databases. Duplicate genes between pathways were removed, and then, 619 genes were identified (Table 1).

### 2.2. TCGA-HCC, GSE14520, and HCCDB18 Data Preprocessing

TCGA-HCC's RNA-Seq data processing is as follows: (1) We eliminated any samples that lacked information about clinical follow-up, survival time, and status. (2) Ensembl was converted to gene symbol. (3) When comparing the expression of various gene symbols, we used the median value. (4) Genes whose expression in sample < 0.5 were filtered, which accounted for more than 50% of the genes.

Dataset processing of GSE14520 is as follows: (1) We retained the samples from the GPL3921 platform. (2) Samples lacking information on clinical follow-up information, survival status, and time were removed. (3) HCC samples were retained. (4) Probes were converted to gene symbol. (5) A probe in response to multiple genes was removed. (6) The expression of multiple gene symbols was assessed by taking its midvalue.

Dataset processing of HCCDB18 is as follows: (1) We eliminated any samples that lacked information on clinical follow-up, survival time, and status. (2) HCC samples were retained. The following are the pretreatment samples: TCGA 365, GSE14520 221, and HCCDB18 203 (Table S1).

### 2.3. HCC Subtype Classification

First, the “metabolic genes” that were linked to prognosis were screened using a univariate Cox regression analysis. By employing the ConsensusClusterPlus technique, the 365 TCGA-HCC samples were clustered, and stable clustering findings were established based on the cumulative distribution function (CDF) as well as the CDF delta area curve. With the help of the specified metabolic genes, the HCC metabolic genes were constructed. The rationality for clustering is validated with the use of the resampling-based technique known as the ConsensusClusterPlus 1.52.0 method. km arithmetic and “1-Spearman correlation” distance were utilized to complete 500 bootstraps with every bootstrap having specimens (≥80%) of TCGA-HCC sample dataset. Cluster number *k* was between 2 and 10, and the optimum *k* was identified as per cumulative distribution function (CDF) and consistency matrix. The process of resampling has the potential to disrupt the original dataset. In this way, cluster analysis was carried out on each of the resampled samples, and the results were then analyzed in detail. The results of the analysis of subclusters provided an evaluation of consistency (consensus).

### 2.4. Single-Sample Gene Set Enrichment Analysis (ssGSEA)

Single-sample GSEA (ssGSEA) is an extension of gene set enrichment analysis (GSEA). The absolute degree to which genes from a particular gene set are enriched in a sample is reflected by the ssGSEA enrichment score assigned to each gene. The levels of gene expression for a particular sample were categorized and then standardized. To compute an enrichment score, the empirical cumulative distribution function (ECDF) was applied to both the genes that were included in the signature and the genes that were retained. We employed the ssGSEA approach to determine each patient's IFN*γ* score to assess the Th1/IFN*γ* expression variations in metabolic subtypes and enrichment score of 14 oxidative stress-related biological pathways. The ssGSEA score was normalized to uniform distribution, for which the ssGSEA score is distributed between 0 and 1.

### 2.5. Immune Infiltration Characteristics

To contrast the immunological properties of the various metabolic groups, when analyzing the score distribution and differential expression of 22 different immune cells present in the TCGA-HCC sample, we made use of the CIBERSORT technique. CIBERSORT [22] is a technology that may be used to deconvolve the expression matrix of different types of immune cells using the linear support vector regression methodology as the foundation. The analysis of the expression pattern based on transcriptomic sequencing was performed with the CIBERSORT tool, and the denoising method and the unknown mixture content were removed by the deconvolution method to determine the relative proportion of each of the 22 distinct types of immune cells. The relative expression of certain genes was assessed based on the data from the expression patterns of each sample that was sequenced. This allowed for the proportions of 22 different types of immune cells to be predicted.

### 2.6. Prediction of Chemotherapeutic/Immunotherapeutic Response and Establishment of the Subtype Characteristic Index

To examine the similarities between patients' diverse metabolic subtypes and the GSE91061 dataset (melanoma dataset undergoing anti-CTLA-4 and anti-PD-1 therapy), we used a subclass mapping technique. When the *p* value is decreased, the degree of similarity increases. At the same time, we compared the degree of responsiveness between various subtypes and conventional chemotherapeutic agents (cisplatin, vinorelbine, embellin, and pyrimethamine) using the same methodology.

We employed linear discriminant analysis, also abbreviated as LDA, to create a subtype classification feature index so that we could more accurately measure the immunological features of patients who were represented by a variety of sample cohorts. In the TCGA dataset, we employed the LDA model to compute each patient's subtype feature index, and we examined the feature index of each of the distinct subtypes. Within the TCGA dataset, we assessed the characteristics that were linked to prognosis. Firstly, a z-score was done on each individual feature, and Fisher's LDA optimization standard was utilized to specify that the centroids of each group should be as dispersed as possible. It was discovered that a linear combination A maximized the between-class variance of A in comparison to the variance of the within-class measure. The properties of the model allow for the differentiation between samples of various subtypes analyzed.

### 2.7. Weighted Correlation Network Analysis (WGCNA)

We clustered the samples using the R software program WGCNA and then filtered the coexpression modules of metabolic genes after selecting the TCGA expression profile dataset with a MAD value of more than 50%. According to the findings of the research, the coexpression network conforms to the scale-free free network; i.e., the logarithm of the connection degree *k* (log(*k*)) of a node has an inverse correlation with the logarithm of the likelihood that the node occurs, which is denoted by log(*p*(*k*)), and the correlation coefficient is >0.85. Further conversion into an adjacency matrix was performed on the expression matrix, and after that, a topological matrix was derived from the adjacency matrix. To cluster genes, we utilized the TOM and a technique called average-linkage hierarchical clustering as per the criteria of the hybrid dynamic shearing tree and established 80 as the basic threshold for each gene count in the gene network module. After identifying the gene modules with the use of the dynamic shear approach, we carried out cluster analysis on the modules before calculating each module's eigengenes in turn. We created a new module by merging the modules that were physically located closer to one another and set minModuleSize = 80, DeepSplit = 3, and height = 0.25.

### 2.8. Statistical Analysis

R (version 3.6.0) was adopted to execute all analyses of statistical data. All statistical tests were bilateral. The statistical significance level was established at *p* value < 0.05.

## 3. Results

### 3.1. Molecular Typing Based on Metabolic Gene Construction

**Figure 1:**
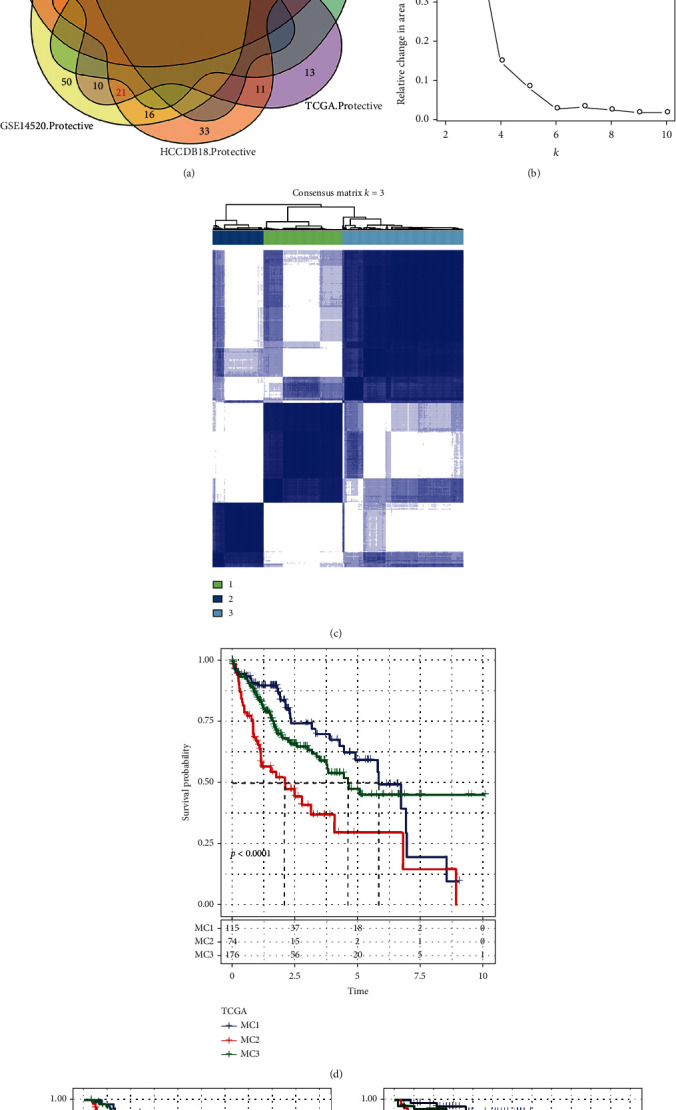
The metabolism cluster in HCC. (a) Intersection Venn diagram showing metabolic genes with substantial prognostic significance in the 3 cohorts. (b) CDF curve of cohort samples from the TCGA and CDF delta area curve, consensus clustering's delta area curve, reflecting the degree of variance in the area under the cumulative distribution function (CDF) curve for each classification number k in comparison to *k*–1. The category number k is represented on the horizontal axis, and the relative change of the area under the CDF curve is shown on the vertical axis. (c) Heat diagram of a sample clustering when consensus *k* = 3. (d–f) KM curve of the prognostic relationship of the three subtypes in the TCGA (d), GSE14520 (e), and HCCDB18 (f) datasets.

To construct molecular subtypes, we performed the univariate Cox regression analysis of metabolic genes in the three datasets, and the results of the intersection with prognostic-related genes (TCGA: 214, GSE14520: 133, and HCCDB18: 169, Table S2-4) showed that there were only 30 prognostic-related genes (Figure 1(a)), which indicates that the consistency of metabolic genes between datasets on different platforms is poor, and the expression of a particular metabolic gene might vary remarkably depending on the cohort. As a consequence, to conduct the subsequent analysis, we based on 30 metabolic genes that were all recognized as prognostic-related genes (*p* < 0.05).

Consensus clustering (ConsensusClusterPlus) was performed on 365 TCGA-HCC samples to determine a stable clustering result (cluster = 3, Figure 1(b)), and lastly, three metabolic subtypes (metabolism cluster, MC1/2/3) were obtained (Figure 1(c)). Additional investigation into its prognostic qualities revealed that the prognosis for patients within MC2 was unfavorable, while the prognosis for those within MC1 was positive, and the disparity between the two was statistically remarkable (Figure 1(d)). In GSE14520 and HCCDB18, we also observed the same result (Figures 1(e) and 1(f)). These findings suggest that the 3 metabolic subtypes that were developed premised on metabolic genes are reproducible across multiple research groups.

### 3.2. The Link between Metabolic Subtypes, Oxidative Stress, and Common Gene Mutations

**Figure 2:**
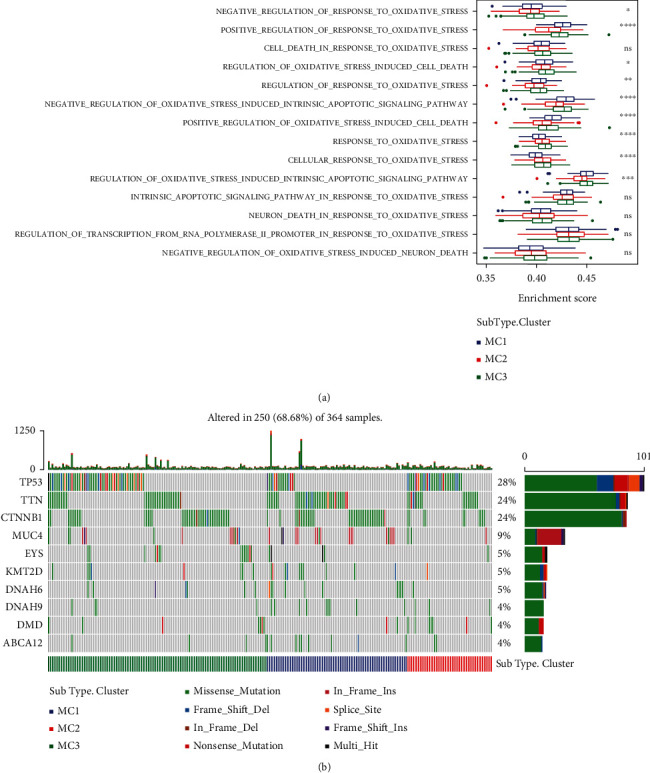
Differential distribution of oxidative stress in different molecular subtypes. (a) Distribution of 14 oxidative stress-related pathways in three subtypes. (b) The variance in the distribution of the total number of gene mutations found in the samples corresponding to the three different molecular subtypes. The *p* value was determined with the use of the rank sum test; ^∗^ is less than 0.05; ^∗∗^ is less than 0.01.

To study the link between metabolic subtypes and oxidative stress, we obtained the TCGA expression profile dataset and then assessed the enrichment scores of 14 oxidative stress-related biological pathways in each sample by using the ssGSEA method. Then, we calculated the distribution of these oxidative stress pathway enrichment scores in the three metabolic molecular subtypes, and we observed that there were significant differences in 9 (64.3%) oxidative stress-related pathways. Among these significantly different biological pathways, except for “response to oxidative stress” and “cellular response to oxidative stress,” MC2 has lower scores in oxidative stress-related pathways (Figure 2(a)). These results indicated that changes in oxidative stress levels are related to different clinical outcomes of hepatocellular carcinoma. Following this, we filtered an additional 2484 genes (mutation frequency > 3) and utilized the chi-square test to search for genes exhibiting considerably greater mutation frequencies in each subtype (threshold value *p* < 0.05), ultimately yielding 133 genes (Figure 2(b)).

### 3.3. Expression of Chemokines in Metabolic Typing and Expression of Immune Checkpoint Genes

**Figure 3:**
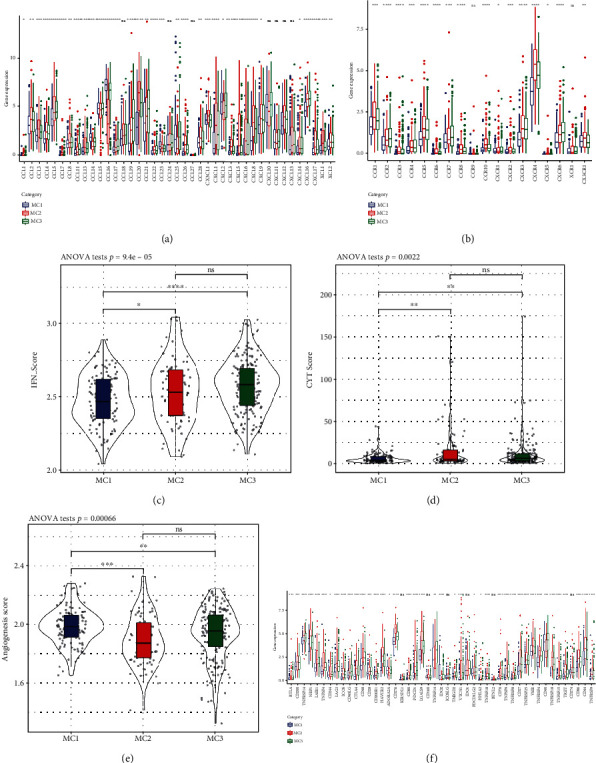
Expression of chemokines and immune checkpoints in different metabolic subtypes. (a) Variation in the distribution and expression of chemokines in TCGA cohort. (b) Variations in the distribution and expression of chemokine receptors in TCGA cohort. (c) Differences in the IFN*γ* score distribution across the various subgroups derived from TCGA cohort. (d) Variations in the lysis activity of immune T cells between the two subgroups. (e) Scores on angiogenesis that were variable across the several groups. (f) Variations in the TCGA cohort's immune checkpoint genes both in terms of their expression and their distribution. Analysis of variance was the statistical method that was used to assess the significance. ^∗^ means *p* < 0.05; ^∗∗^ means *p* < 0.01; ^∗∗∗^ means *p* < 0.001; ^∗∗∗∗^ means *p* < 0.0001.

To examine the different ways in which chemokines are expressed among the 3 different metabolic subtypes, we evaluated the expression of chemokines as well as their corresponding receptor genes in the TCGA cohort. Among 41 chemokines, 34 (82.93%) had substantial variations in subtypes (Figure 3(a)), while 16 of the 18 chemokine receptor genes (88.89%) exhibited substantial variations in the expression of metabolic subtypes (Figure 3(b)). From these findings, it is convinced that various metabolic subtypes will have a varying degree of immune cell infiltration.

We obtained Th1/IFN*γ* gene signatures to assess the variations in expression levels of Th1/IFN*γ* that exist across the 3 metabolic categories [25], and by using ssGSEA methodology, we determined each patient's IFN*γ* score. According to the findings, the MC2 and MC3 subgroups both exhibited greater IFN*γ* scores, whereas the MC1 subgroup exhibited the smallest IFN*γ* scores (Figure 3(c)).

To examine the lytic activity of immune T cells in relation to the 3 metabolic subtypes, we used the average expression levels of GZMA and PRF1 [26] to determine the level of lytic activity exhibited by immune T cells in each patient's tumor. Interestingly, MC2 and MC3 possessed the greatest immune T cell lysis activity, whereas MC1 possessed the least, and there was a significant difference between subgroups (Figure 3(d)).

To examine the variances in angiogenesis score expression between the three metabolic subgroups, we obtained the angiogenesis-related gene set [27] to analyze each patient's angiogenesis score. The findings illustrated that MC1 had a considerably greater angiogenesis score as opposed to MC2 and MC3. The difference between subgroups was significant (Figure 3(e)).

47 immune checkpoint-related genes were analyzed to determine the expression variations across the 3 metabolic categories [25]. The results illustrated that 41 (87.23%) genes were present in the 3 metabolic subgroups. There were remarkable variations among the subtypes. It was found that the expression of the majority of genes associated with immune checkpoints was much higher in MC2 cells in contrast with that in MC1 cells. The levels of expression of T cell exhaustion markers including HAVCR2, CD276, PDCD1, CTLA4, and LAG3 were much greater in MC2 cells as opposed to the levels in MC1 cells (Figure 3(f)). Based on these findings, it seems that distinct patient subgroups might exhibit varying degrees of immunotherapeutic response.

### 3.4. Immune and Metabolic Pathway Features in Various Metabolic Subgroups

**Figure 4:**
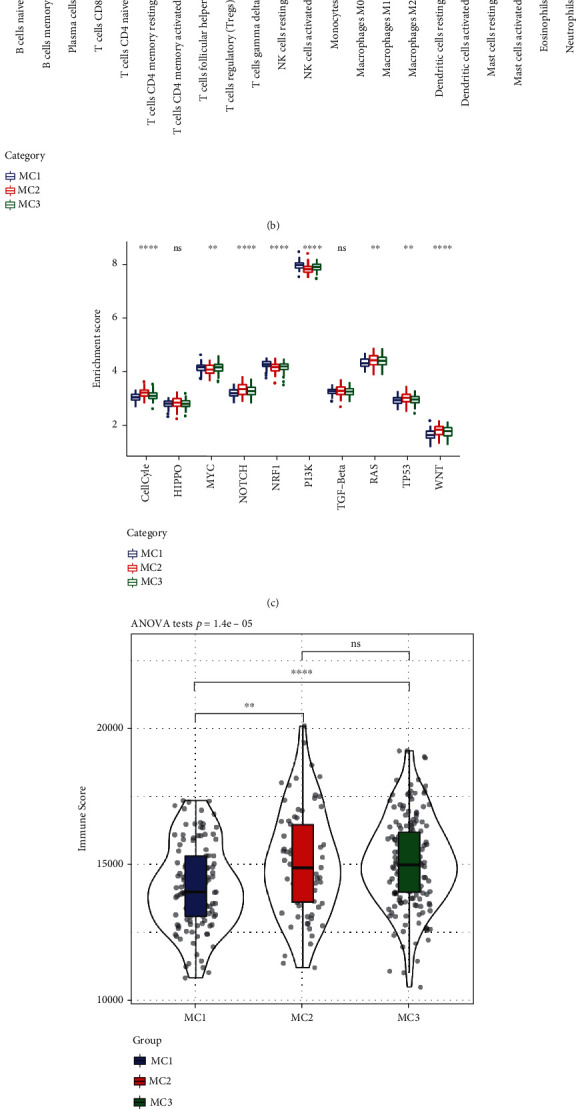
Immune and pathway characteristics in different metabolic subtypes. (a) The ratio of each of the 22 distinct immune cell components found in the samples across the various subgroups. (b) Variations in 22 immune cell components between samples belonging to various groups. (c) Variations in the scores of ten pathways associated with aberrations in tumors observed in various subgroups. (d) Scores for immune infiltration varied significantly among the various groups. (e) An examination of the similarities and differences between the 3 metabolic molecular subtypes and the previous six pan-cancer metabolic molecular subtypes.

We adopted CIBERSORT to assess the immune properties in distinct metabolic subtypes. According to the findings, there were remarkable variations in the immunological features shown by each of the subtypes (Figure 4(a)). Different CD8 T cells, M0, M1, and M2 macrophages as well as resting CD4 memory T cells were significantly and highly expressed in different subtypes (Figure 4(b)), indicating that they may have an instrumental function in HCC.

We examined 10 distinct oncogenic pathways to ascertain their characteristics across various metabolic subtypes [28]. According to the findings, eight of the ten pathways displayed substantially different characteristics depending on the subtype. Among these 8 pathways, MC2 scores were relatively high in the cell cycle, NOTCH, RAS, and TP53 pathways, while the remaining two pathways had low enrichment scores in MC2 (Figure 4(c)). The results of an analysis of immune infiltration revealed that MC2 and MC3 had greater levels of immune microenvironment infiltration compared to MC1, and MC1 was shown to have the least ImmuneScore (Figure 4(d)).

We acquired the data on molecular subtypes [29] from these samples to examine the link between these molecular subtypes and the previous six pan-cancer immunotypes. The findings demonstrated that there are substantial variations across the various pan-cancer immunotypes (Figure 4(e) and Figure S1). The C1 and C2 subtypes with unfavorable prognosis accounted for significantly higher proportions in our definition of MC3 and MC2 subtypes than MC1, and the proportion of C3 with better prognosis in MC2 was significantly lower than MC1 (Figure 4(e)), which is consistent with the poor prognosis of MC2 and MC3. Based on these outcomes, it appears that these 3 subtypes could be added to the established six subtypes that were employed in the prior research.

### 3.5. Analysis of the Variation in TIDE across Metabolic Subtypes

**Figure 5:**
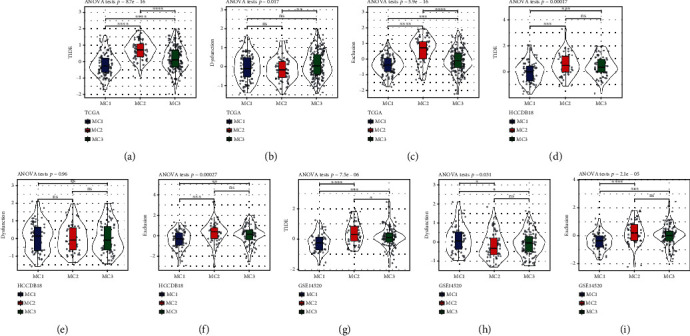
Differential analysis of TIDE in various metabolic subtypes. (a–c) TCGA score difference of TIDE, T cell rejection, and T cell dysfunction in various metabolic subtypes. (d–f) HCCDB18 score difference of TIDE, T cell rejection, and T cell dysfunction in distinct metabolic subtypes. (g–i) GSE14520 score difference of TIDE, T cell rejection, and T cell dysfunction in various metabolic subtypes.

We utilized the TIDE (http://tide.dfci.harvard.edu/) tool to assess the possible clinical impacts of immunotherapy in the three metabolic subtypes. The greater the TIDE prediction score, the greater the likelihood of immune evasion, suggesting that immunotherapy is less likely to be effective for the patient. The results showed that the TIDE score of MC2 in the TCGA dataset was considerably elevated in contrast with that of MC1 (Figure 5(a)), indicating that MC1 is more likely to respond favorably to immunotherapy as opposed to MC2/3. Moreover, by comparing the findings of the T cell dysfunction versus rejection scores, we discovered that MC2 displayed a lower T cell dysfunction score in contrast with MC1/3 (Figure 5(b)); MC2 was shown to have an elevated T cell rejection score, whereas MC1 had a higher score (Figure 5(c)). We observed the same results in the HCCDB18 (Figures 5(d)–5(f)) and GSE14520 (Figures 5(g)–5(i)) datasets. These findings could explain why the prognosis for MC2 is unfavorable while that of MC1 is satisfactory.

### 3.6. Comparative Analysis of Metabolic Subgroups and Chemotherapy/Immunotherapy

**Figure 6:**
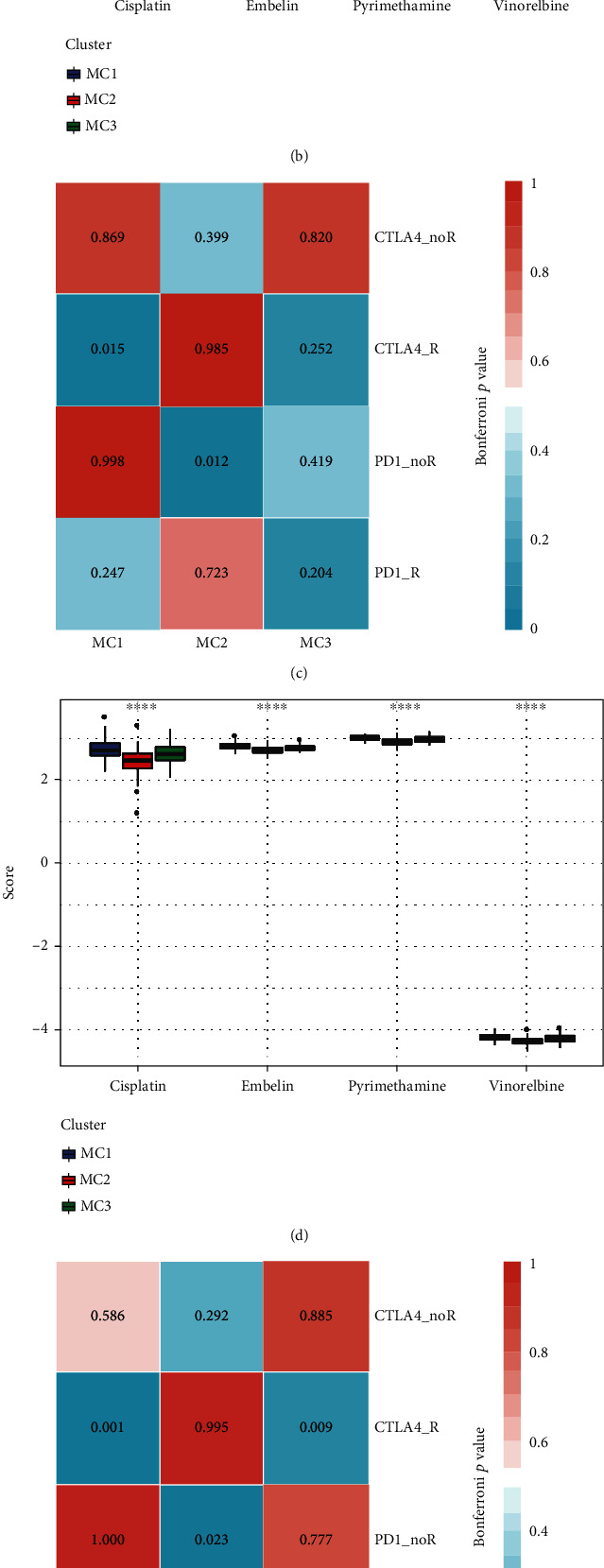
Differential expressions of immunotherapy/chemotherapy in various metabolic subtypes. (a, b) According to the results of the TCGA submap study, MC1 might be more responsive to CTLA4 (Bonferroni-corrected *p* < 0.05). Box plots of the estimated IC50 for MC1/2/3. (c, d) The results of the HCCDB18 submap analysis suggested that MC1 might be more responsive to CTLA4 (Bonferroni-corrected *p* < 0.05). Box plots of the estimated IC50 for MC1/2/3. (e, f) As per the findings of the GSE14520 submap analysis, MC1 might be more responsive to CTLA4 (Bonferroni-corrected *p* < 0.05). Box plots of the estimated IC50 for MC1/2/3.

To assess the variations between immunotherapeutic and chemotherapeutic interventions in various metabolic subtypes, we compared the similarities across the 3 metabolic subgroups and immunotherapy patients in the GSE91061 dataset using subclass mapping. We found that in different datasets, the MC1 subtype showed a greater degree of sensitivity to CTLA4 inhibitors compared to the other two subtypes (Figures 6(a), 6(c), and 6(e)). When we compared the responses of various subtypes to standard chemotherapeutic agents (cisplatin, vinorelbine, embellin, and pyrimethamine), we discovered that the MC2 subtype was more responsive to these four medications (Figures 6(b), 6(d), and 6(f)).

### 3.7. Establishment of the Metabolic Subtype Characteristic Index

**Figure 7:**
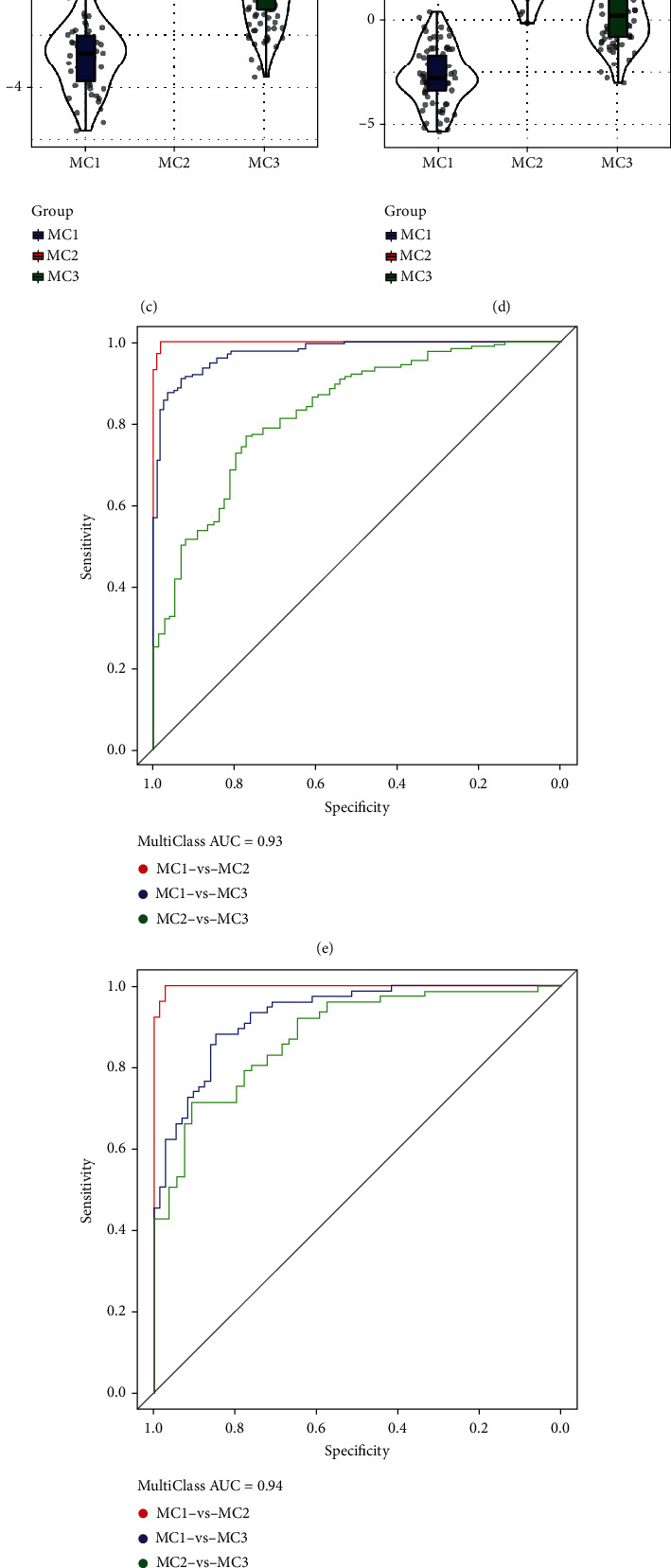
Immune characteristic index and ROC curve in various metabolic subtypes. (a) The link between the first two characteristics of the TCGA metabolic feature index and the different subtypes of metabolism. (b–d) TCGA, GSE14520, and HCCDB18 datasets of different subtypes of immune feature index differences. (e–g) TCGA, GSE14520, and HCCDB18 dataset immune characteristic index ROC curve.

We utilized linear discriminant analysis (LDA) to construct a subtype classification feature index premised on 30 prognosis-related parameters which allowed for the accurate assessment of the immunological characteristics of patients in various sample cohorts. The first two characteristics of the model may be differentiated as samples of several subtypes (Figure 7(a)). We noticed statistically significant variations between the characteristic indices of the various subtypes when using the LDA model to generate each subtype's value for its subtype characteristic index (Figure 7(b)). ROC analysis illustrated that the characteristic index was in different subtypes. For the classification performance in the model, the multicategory comprehensive prediction AUC was 0.93 (Figure 7(e)). The metabolic subtype feature index was applied to the GSE14502 and HCCDB18 datasets, and the results were similar to the TCGA dataset: the feature indexes of distinct subtypes varied significantly (Figures 7(c) and 7(d)), and ROC analysis illustrated that the comprehensive AUC was 0.94 and 0.93, respectively (Figures 7(f) and 7(g)). Those data suggested that those 3 MCs had better predictive ability.

### 3.8. Determination of Metabolic Feature Index among Coexpressed Gene Modules

**Figure 8:**
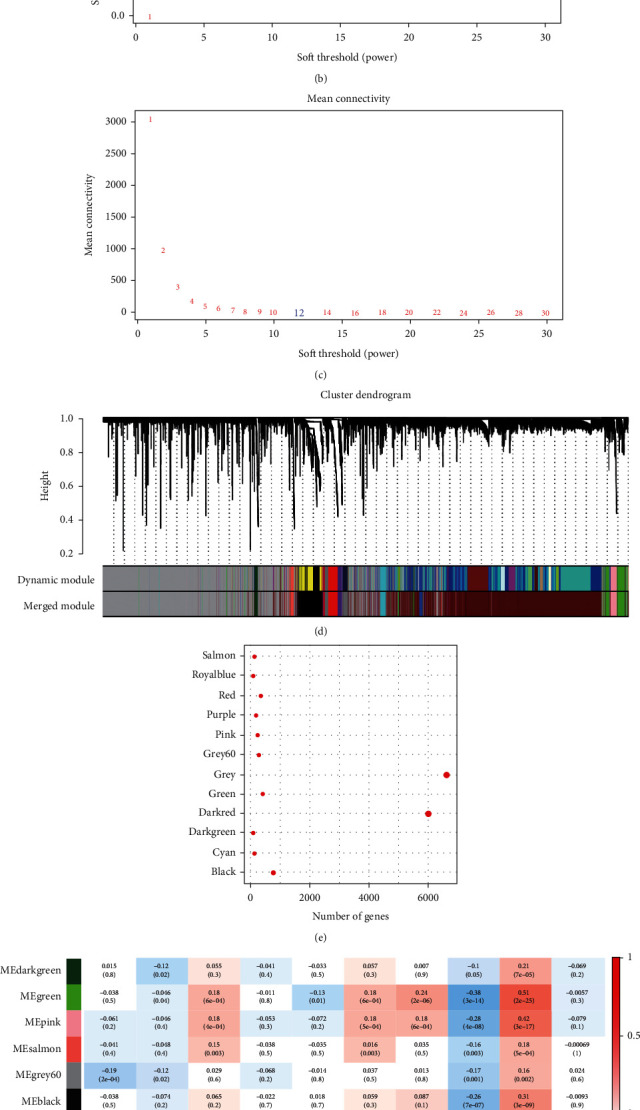
Establishment of metabolic index coexpressed gene module. (a) A tree representing each sample's clustering. (b) An investigation of the scale-free fit indices for several different soft-thresholding powers (*β*). (c) An examination of the average degree of connectivity for several different soft-thresholding powers. (d) A dendrogram showing the clustering of all genes and lncRNAs with differential expression according to a dissimilarity measure (1-TOM). (e) Statistical information on the proportion of genes included in each module. (f) The clinical information corresponds to each module's content.

We clustered the samples with the help of the WGCNA package of the R tool and screened the coexpression modules (Figure 8(a), soft threshold = 12). To guarantee that the network was not susceptible to scaling problems, we chose *β* = 12 and obtained 12 modules (Figures 8(b)–8(d)). The gray module represents a set of genes that cannot be merged with those of other modules.  Figure 8(e) displays the data on the transcripts of each individual module. Gene modules that cannot be assigned are described as gray modules. Each module's relationship to the patient's age, sex, M stage, N stage, T stage, stage, MC1, MC2, and MC3 was examined. The module had a substantial and negative correlation with MC1 and a significant and positive correlation with MC2, respectively. The correlation between the green, pink, and salmon modules and MC2 was greater than 0.4 (Figure 8(f)).

### 3.9. Prognostic Analysis of Metabolic Feature Index in Coexpressed Gene Modules

**Figure 9:**
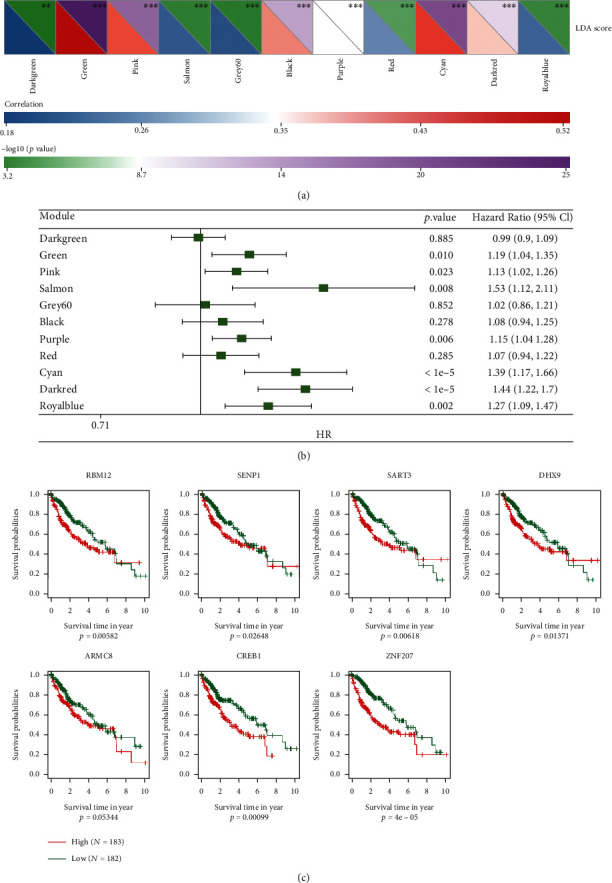
Key genes identified in dark module. (a) An examination of the relationship between the LDA score and the metabolic feature index. (b) Correlational analysis of prognostic factors linked to the immune feature index. (c) Prognostic KM analysis of key genes.

We determined the degree to which these 12 modules' feature vectors were correlated with the metabolic feature indices by performing correlation analysis. According to the findings, each of the 12 modules had a significant correlation with the immunological characteristic index (Figure 9(a)). To conduct a reliable and accurate prognostic analysis, we chose modules that had a strong correlation with the metabolic feature index, and we observed that green, purple, cyan, dark, salmon, and royal blue were significantly correlated (Figure 9(b), *p* ≤ 0.01). We further screened the green and dark modules based on the link between the module, metabolic molecular subtypes, and prognosis, with the module feature vector correlation coefficient > 0.9 and the prognostic significant gene (*p* < 0.01) as the hub genes for the module. Finally, seven key genes (*RBM12*, *SENP1*, *SART3*, *DHX9*, *ARMC8*, *CREB1*, and *ZNF207*) were identified in the dark module. At the same time, we classified the patients into groups with low and high expression premised on gene expression and evaluated whether there were prognostic differences between the genes in these two expression groups. The findings illustrated that except for the group survival curve of the ARMC8 gene, which was marginally significant, the survival curves of other genes had significant differences (Figure 9(c)).

Next, we enriched the genes of the dark and green modules (cluster profile package). The findings highlighted that the dark module was associated with tumor processes, including DNA replication, cell cycle, and autophagy-animal (Figure S2). Meanwhile, the green module was related to Huntington's disease, Parkinson's disease, Alzheimer's disease, and other related diseases (Figure S3).

## 4. Discussion

Currently, clinical decisions for HCC are often based on the disease staging system. It divides patients into different subgroups according to clinical factors associated with HCC prognosis, especially pathological factors, and determines the corresponding treatment plan [30]. Clinical factors associated with the prognosis of patients with HCC mainly reflect the degree to which tumors have spread (distant metastasis, vascular invasion, number of tumor nodules, tumor size, and others) and the severity of liver damage (symptoms of liver decompensation, protein synthesis, and detoxification function, among others) [31]. Although this classification method based on pathological diagnosis has effectively guided clinical practice and brought tangible benefits to patients with HCC, it can only describe the biological characteristics of tumors at the tissue level and fails to accurately represent the biological nature of tumors, especially the essential differences in molecular biology between different classifications [32]. In addition, considering that HCC is such a very heterogeneous illness, even individuals with the same TNM stage might have substantially different responses to therapeutic drugs and varying duration of survival time.

With the in-depth analysis of the molecular pathological characteristics of HCC, some studies have explored the link between the expression profiles of specific genes and the clinicopathological characteristics of HCC, opening new doors for clinical practice. However, it is difficult to attain a global comprehension of the molecular biological characteristics of HCC metastasis, recurrence, and prognosis based on the expression level of one or a few genes. With the development of molecular pathology research on tumors genome-wide, a new tumor typing method, tumor molecular typing, has emerged. It classifies tumors according to their pathological characteristics (mainly genomic characteristics) at the molecular level. Molecular typing can provide global features at the molecular level, such as the tumor genome/proteome/metabolome, enrich molecular pathological information on tumor occurrence and progression, and provide support for clinical diagnosis, staging, individualized treatment, and prognosis prediction [33].

In the current research, we attempted to molecularly categorize HCC at the metabolic level, and as a result, we discovered some novel insights. Our samples were classified into three different metabolic subtypes (MC1, MC2, and MC3) premised on the 619 metabolic genes that were used to classify HCC, where the subtypes showed significant differences in prognosis (Figure 1). The immunological properties of the various metabolic subtypes were distinct, and these subtypes might have varied responses to immunotherapy (Figure 3). In independent datasets (HCCDB18 and GSE14520), there was a significant degree of reproducibility across metabolic subtypes. To more accurately assess the immunological characteristics of patients and accurately represent their varying degrees of immune infiltration, an immune feature index was developed premised on the metabolic subtypes. There was a link between the metabolic feature index and the immune checkpoint. In the meantime, using the coexpression network analysis as a foundation, we conducted a screening of seven possible gene markers (*RBM12*, *SENP1*, *SART3*, *DHX9*, *ARMC8*, *CREB1*, and *ZNF207*) that are linked to the metabolic feature index.

The TME has been shown to have a significant modulatory function in the onset and advancement of tumors. The TME that is generated as a result of the process of dynamic alterations is controlled by a wide range of immunosuppression signals, and its diversity may help determine several factors, particularly patients' prognosis and how well they will respond to therapy [34, 35]. Recently, numerous studies have discovered that the onset and growth of malignant tumors are directly linked to the components that are present in the microenvironment that surrounds tumor cells. For instance, the chemokines and cytokines that are produced in the liver can enhance angiogenesis as well as immune evasion and antiapoptotic responses and may activate a range of immune cells inside the TME, assist T cells in entering the tumor, alter the immune response of the tumor, and mediate the therapeutic benefits of the treatment [36]. According to the findings of our investigation, the expression of chemokines and the genes that code for their receptors is significantly distinct across various metabolic subtypes. These differential expressions suggest that various metabolic subgroups have varying levels of immune infiltration, which might influence the progression of tumors and patients' immune response to them. Furthermore, tumor-associated chemokines and cytokines may mobilize and polarize immunological subpopulations as well as facilitate the differentiation of cells into protumor phenotypes, thus promoting the progression of tumors. Tumor-associated macrophages (TAMs) are among these immune subtypes and can be polarized to M2 by IL-13, IL-4, TGF-*β*, or IL-10, within the TME. Macrophages have a phenotype and are responsible for the growth of tumors. Additionally, they stimulate angiogenesis, which is necessary for the recruitment of regulatory cells (Tregs) [37]. In most cases, an unfavorable prognosis for a variety of cancers, including HCC, is linked to the accumulation of TAMs in the area of the tumor.

Long-term strong exposure to reactive oxygen species can induce chronic inflammatory disease progression and carcinogenesis [38, 39]. ROS has been shown to be associated with cancers of the digestive system, such as gastrointestinal cancer, cholangiocarcinoma, pancreatic cancer, and HCC [40, 41]. Representative mechanisms of HBV- and HCV-related chronic liver disease progression and hepatocellular carcinoma have been shown to involve the function of viral proteins, such as immune interference, tumor initiation or tumor suppression interference, and oxidative stress response induction [42]. In Figure 2(a), several oxidative stress-related pathway scores were different in 3 MCs.

We observed that CD4+ T cells, macrophages, and CD8+ T cells are expressed at a high level in various metabolic subtypes. Although the presence of cells such as Tem and Trm is linked to the prognosis of patients with HCC, these cells commonly express PD-1. Immune depletion markers including cytotoxic T lymphocyte-associated antigen-4 (CTLA-4) and lymphocyte activation gene 3 (LAG-3) are inversely related to their function [43]. Therefore, these depletion markers have become the primary targets for the immune checkpoint blockade (ICB) to activate and restore CD8+ T cell function [44]. Moreover, CD8+ T cells within the TME may synthesize IFN-*γ*, thereby promoting the upmodulation of IDO1 and PD-1/PD-L1 gene expression [45]. Research has illustrated that PD-L1 upregulation in tumor cells, particularly when combined with PD-1 produced by tumor-infiltrating activated T cells, may cause exhaustion and suppress the antitumor immunological function of these effector cells, which allows tumor cells to escape the immune system [46]. Additionally, the density of CD8+ T cells with high inhibitory expression of PD-1 is linked to a grim clinical prognosis for HCC [47]. IDO1 upregulation has been shown to have a favorable correlation with not only a dismal prognosis but also tumor advancement and metastasis [48, 49].

These existing research results have also been confirmed in our study. Our research evaluated the Th1/IFN*γ* ratio, immune checkpoint-related genes, angiogenesis score, and immune T cell lytic activity in the 3 metabolic subtypes. Combined with the above findings, we discovered that MC2 had a high score for immune T cell lysis activity (Figure 3(d)), implying that this subtype possessed more potent immunogenicity and a suitable TME, which should bring better clinical results, but its prognosis was worse than that of MC1. In the comparative study of the expression of immune checkpoints in various subtypes, we discovered that compared to that in MC1, the level of expression of the majority of genes associated with immunological checkpoints (HAVCR2, CD276, PDCD1, CTLA4, and LAG3) was much higher in MC2, suggesting that MC2 may be exhausted by T cells in the subtypes, which might explain why MC2 had an elevated immune microenvironment infiltration level but also an unfavorable prognosis.

In 2017, the TCGA team analyzed the histopathological data of 196 cases of HCC and showed that 22% of HCC exhibited moderate or high lymphocyte infiltration; the team further analyzed the gene expression results of 66 immune markers [3]. Unsupervised hierarchical clustering identified 6 tumor sample clusters, two of which showed high expression of immune markers, including the following immune checkpoint genes: cytotoxic T lymphocyte-associated protein 4 (CTLA4), procedural death receptor 1 (PD-1), and programmed death ligand 1 (PD-L1). This may indicate that ICI treatment can substantially affect HCC with moderate or high lymphocyte infiltration and high expression of immunosuppressive molecules [50]. Combined with our research, it is suggested that MC2 patients may have excellent responsiveness to ICI treatment during the treatment of HCC. Furthermore, we screened 7 potential genetic markers. To date, many studies have confirmed that multigene markers have good predictive power for the metastasis, progression, recurrence, and survival rate of HCC [51, 52]. Gene signatures are a potentially useful high-throughput molecular identification tool via their use in clinical practice, which is premised on gene expression profiling. In short, this sort of combination model based on clinical, pathological, and genetic traits has been proven by accumulating research as having the potential to be highly applicable in clinical settings [53].

There is accumulating evidence to suggest that epigenetic alterations perform an instrumental function in carcinogenesis. Epigenetic alterations have been linked to the clinical prognosis of HCC patients by several different research investigations. This also complicates the molecular classification of HCC. These findings expand the potential therapeutic targets of HCC and enable us to have deeper insights into the molecular classification of HCC by integrating this aspect of the molecular characteristics. In the future, we plan to conduct experimental verification of the results of this article and use more reasonable bioinformatic strategies to improve the model. In addition, the HCC population has undergone tremendous changes in the past few years. The proportion of HCC and early HCC caused by nonhepatitis viruses is gradually increasing [54]. Research on HCC should pay more attention to this part of the population's molecular characteristics and molecular typing.

In summary, the results of this research established a metabolic classification that is capable of independently functioning as an HCC prognostic indicator of HCC. It measured the HCC patients' prognosis risk by analyzing the differences in the features of the TIME across subtypes in order to help clinical diagnosis, staging, and personalized therapy. In addition, prognostic prediction can be used to provide support to HCC patients.

## Figures and Tables

**Table 1:** Metabolism-related genes.

All metabolism-related gene list								
ACSS2	ACYP2	VCAN	B4GALT2	PHKG2	EXT1	CYP2D6	GLYAT	AKR7L
GCK	ME1	PHKA2	B4GALT6	B3GNT7	BGN	CYB5R3	MAT1A	GSTM2
PGK2	ACYP1	TPR	PPP1R3C	SEC13	HS3ST4	GSTZ1	CYP2U1	SULT1A4
PGK1	ME2	HEXB	GOT1	ST3GAL2	SLC25A10	GSS	NAT2	UGT2B11
PDHB	MDH2	GYG2	NUP43	SLC35B2	GPC6	AHCY	UGT2B4	AS3MT
PDHA1	MDH1	NDC1	B4GALT4	ACAN	CHST6	CYB5B	N6AMT1	ACSM4
PDHA2	PC	GPC1	FMOD	B4GALT5	B3GALT5	IMPAD1	GLYATL2	CYP21A2
PGM2	GLO1	ST3GAL6	HS3ST2	PFKFB1	PGP	SULT2A1	CBR3	UGT1A5
TPI1	ACACB	PHKA1	CHST3	B4GALT3	HS6ST3	CYP3A5	CYP3A4	UGT1A9
ACSS1	ACACA	HYAL2	PFKFB2	ADPGK	HYAL3	AHR	CYP3A7	UGT1A1
FBP1	AKR1B1	PYGM	CHPF	SLC37A1	RHD	CYP2C18	CYP11B1	UGT1A8
ADH1B	ACOT12	NUP133	SDC4	G6PD	CHP1	UGT2B10	FDXR	UGT1A10
HK2	CPT1A	NDST1	SLC35B3	ST6GALNAC6	NHLRC1	SULT1E1	AKR7A3	UGT1A3
ADH1C	CPT1C	PRKACA	NUP153	HS3ST6	AGRN	UGDH	BPNT1	GSTA1
HK1	ACADS	HMMR	GOT2	SDC3	RHCE	AIP	HPGDS	ACY1
HK3	ACADSB	NUP37	HS3ST3B1	B3GALT2	PRELP	PTGES3	CMBL	GSTA2
ADH4	ACADL	GPC4	NUP85	AGL	POM121	CYP27B1	CYP2C19	UGT1A7
PGAM2	ACADM	GCKR	ST3GAL3	NUP35	FUT4	PODXL2	NNMT	UGT1A4
ADH5	CYP4A11	SEH1L	NUP214	GNPDA2	FUT11	AADAC	ACSM1	CYP2A6
PGAM1	ACADVL	B4GALT1	OMD	GLB1L	SHPK	ABHD14B	GLYATL1	SULT1A3
ADH1A	ACAA2	SLC9A1	IDUA	GYG1	RPE	POMC	UGT1A6	FDX2
ALDOC	HADH	NUP50	NCAN	TKT	CHSY3	SLC35D1	DPEP2	UGT2A2
ALDH7A1	HADHB	XYLB	AC010618.1	NDST3	CALM1	MTR	CYP2S1	GSTT1
LDHAL6B	HADHA	AAAS	FUT5	PHKG1	PAPSS2	2-Mar	GGT6	PDK4
PKLR	CYP4A22	NUP188	SLC35D2	PRKACG	NUP62	PTGIS	CYP7A1	PDK2
LDHAL6A	ACSL6	SLC25A1	CHSY1	ALDH1A1	GPC2	MTRR	UGT3A2	MPC1
ENO1	ECHS1	PYGL	NUP210	HGSNAT	HEXA	NQO2	GSTM4	CS
PKM	ACSL5	PYGB	BCAN	PGM2L1	GALT	POR	MAT2A	IDH3G
PFKP	EHHADH	RAE1	LYVE1	CRYL1	UBA52	TPST2	TPST1	PDK3
BPGM	GCDH	PRPS2	SLC37A2	NDST2	PRPS1L1	CHAC1	GSTA4	SDHA
PCK2	ACOX3	PHKB	GNS	B4GALNT2	RPEL1	SULT4A1	UGT2B7	L2HGDH
PCK1	ACSL1	NUP93	CHST5	LALBA	B3GALT4	CYP2E1	NAT1	PDPR
ALDH1B1	ACAA1	HAS3	STAB2	GLYCTK	HS3ST5	GGT7	CYP4F11	FH
ALDH2	CPT2	XYLT1	CHST12	CHST14	POM121C	AOC3	CYP4F22	SLC16A8
ALDH3A1	CPT1B	CEMIP	NUPL2	SDC2	SLC37A4	AOC2	ARNT2	ACO2
AKR1A1	ACOX1	PPP2CB	HS6ST1	DCXR	GCLC	ACY3	CYP7B1	IDH3B
FBP2	ECI2	MAN2B1	PPP2R1B	UGP2	CYP51A1	CNDP2	CES3	OGDH
PFKM	ECI1	GYS1	KHK	CSGALNACT2	AOC1	GSTT2B	CES2	PDHX
PFKL	ACSL3	HAS1	GLCE	GUSB	CYP26B1	GSTM1	TRMT112	PPARD
LDHC	ACSL4	PPP2R1A	NDST4	GLB1	GGCT	GSTM5	SULT1B1	NNT
GAPDH	ACOT4	GAPDHS	NUP54	UBB	FMO3	GSTM3	UGT2A1	SDHB
ENO3	TECR	OGN	PAPSS1	B3GNT2	MGST1	CYP2J2	GSTA3	DLST
ENO2	BAAT	GALK1	LUM	PFKFB3	FMO1	UGT2A3	OPLAH	SUCLA2
PGAM4	ELOVL5	SLC25A11	KERA	HAS2	NR1H4	UGT2B28	CYP11B2	SLC16A3
ADH7	ELOVL6	NUP88	NUP58	HS6ST2	DPEP1	CYP27A1	CYP8B1	MPC2
ADH6	ACOT2	NAGLU	SORD	FUT3	CYP24A1	BPHL	GSTA5	SDHC
LDHB	YOD1	MANBA	MAN2C1	RBKS	CYP3A43	TPMT	ACSM5	ADHFE1
ALDH1A3	ELOVL2	B3GAT1	GALNS	GAA	GCLM	FDX1	ACSM2A	PDK1
ALDH3B1	FADS1	ST3GAL4	G6PC3	CHST11	CYP46A1	CYP19A1	CYP2R1	SLC16A1
ALDH3B2	FADS2	NUP98	PGD	DSEL	MAT2B	CYP1B1	CYP4F2	SUCLG1
ALDH9A1	PECR	NUP107	HSPG2	B3GALT1	AKR7A2	SULT6B1	CYP4F12	PDP1
ALDH3A2	SCD5	GYS2	PRKACB	FUT9	TBXAS1	CYP2C9	1-Mar	IDH3A
GALM	SCD	DSE	RPS27A	FUT10	AHRR	CYP2C8	RXRA	BSG
ALDOA	HACD1	UST	SLC26A1	HPSE2	GSTO2	ESD	CYP4F8	SUCLG2
DLD	ACOT1	B3GAT2	CHST7	HPSE	ACSM2B	NCOA2	CYP4F3	PDP2
DLAT	ACOT7	EPM2A	PRPS1	CSPG4	MAOB	CYP11A1	CYP26C1	FAHD1
ALDOB	HSD17B12	PPP2R5D	GPC3	B4GAT1	CYP2W1	CYP1A1	MAOA	D2HGDH
G6PC2	HACD2	ARSB	CSGALNACT1	FUT1	GSTP1	CYP1A2	SULT1A1	IDH2
LDHA	HS3ST1	GNPDA1	TKFC	CHST2	NCOA1	DPEP3	UGT2B15	SDHD
G6PC	SLC25A13	NUP155	B3GAT3	CHST1	MGST2	CYP4B1	SULT1A2	VDAC1
PGM1	ST3GAL1	PPP2CA	UBC	B3GALT6	SULT2B1	MGST3	CYP2B6	
GPI	IDS	PFKFB4	EXT2	B3GNT4	SMOX	ARNT	CYP2F1	
ME3	DCN	HYAL1	RANBP2	FUT2	COMT	EPHX1	GSTK1	
GRHPR	MAN2B2	GBE1	RPIA	TALDO1	FMO2	CHAC2	CYP2A13	
HAGH	XYLT2	CSPG5	HS2ST1	GPC5	PTGS1	ABHD10	UGT2B17	
ACAT2	DERA	ABCC5	HS3ST3A1	B3GNT3	CYP26A1	CYP4V2	SULT1C4	
ACAT1	CD44	SLC25A12	CHST9	FUT7	HSP90AB1	UGT3A1	CYP2A7	
LDHD	B4GALT7	SDC1	NUP205	CHST13	GSTT2	CYP39A1	SULT1C2	
HAGHL	NUP160	GALE	SLC26A2	SGSH	GGT5	PAOX	CES1	
	CHPF2	SLC2A1	FUT6	CHST15	GGT1	GSTO1	GLYATL3	

## Data Availability

The datasets analyzed in this study could be found in GSE14520 at https://www.ncbi.nlm.nih.gov/geo/query/acc.cgi?acc=GSE14520, in GSE91061 at https://www.ncbi.nlm.nih.gov/geo/query/acc.cgi?acc=GSE91061, and in GSE14502 at https://www.ncbi.nlm.nih.gov/geo/query/acc.cgi?acc=GSE14502.

## Abbreviations

MC: Metabolic subtype
HCC: Hepatocellular carcinoma
TME: The tumor microenvironment
TIME: Tumor immune microenvironment
DEGs: Differentially expressed genes
GEO: Gene Expression Omnibus
CDF: Cumulative distribution function
ssGSEA: Single-sample GSEA
GSEA: Gene set enrichment analysis
ECDF: Empirical cumulative distribution function
LDA: Linear discriminant analysis
WGCNA: Weighted correlation network analysis
TMB: Tumor mutation burden
TAMs: Tumor-associated macrophages
LAG-3: Lymphocyte activation gene 3
CTLA-4: Cytotoxic T lymphocyte-associated antigen-4
PD-1: Procedural death receptor 1
PD-L1: Programmed death ligand 1
ICB: Immune checkpoint blockade.

## Additional Points

*Access to Resources and Materials*. The dataset utilized and processed for this investigation is accessible upon valid request from the corresponding authors.

## Supplementary Materials

Supplementary 1 Supplementary Table S1: clinical characteristics in TCGA/GSE14520/HCCDB18 datasets.

Supplementary 2 Supplementary Table S2: prognosis genes in GSE14520.

Supplementary 3 Supplementary Table S3: prognosis genes in HCCDB18.

Supplementary 4 Supplementary Table S4: prognosis genes in TCGA.

Supplementary 5 Supplementary Figure S1: there were remarkable variations in the pan-cancer immunotypes.

Supplementary 6 Supplementary Figure S2: the dark module was related to tumor processes. (a) Biological process; (b) cellular component; (c) molecular function; (d) KEGG pathway.

Supplementary 7 Supplementary Figure S3: green module was related to nervous system disease. (a) Biological process; (b) cellular component; (c) molecular function; (d) KEGG pathway.
