# Supplementary material for: Development of Prognostic Features of Hepatocellular Carcinoma Based on Metabolic Gene Classification and Immune and Oxidative Stress Characteristic Analysis
Source: Oxid Med Cell Longev. 2023 Feb 18;2023:1847700. doi: 10.1155/2023/1847700 (PMC9969974; doi:10.1155/2023/1847700)
Supplement: Supplementary 1 — Supplementary Table S1: clinical characteristics in TCGA/GSE14520/HCCDB18 datasets. [file 1847700.f1.pdf]

**Table S1. Clinical Features of datasets**

| Clinical Features | TCGA-LIHC | GSE14520 | HCCDB18 |
|---|---|---|---|
| **OS** | | | |
| Alive | 235 | 136 | 168 |
| Dead | 130 | 85 | 35 |
| **T Stage** | | | |
| T1 | 180 | | 33 |
| T2 | 91 | | 96 |
| T3 | 78 | | 59 |
| T4 | 13 | | 15 |
| TX | 3 | | |
| **N Stage** | | | |
| N0 | 248 | | |
| N1 | 4 | | |
| NX | 113 | | |
| **M Stage** | | | |
| M0 | 263 | | |
| M1 | 3 | | |
| MX | 99 | | |
| **Stage** | | | |
| I | 170 | 93 | |
| II | 84 | 77 | |
| III | 83 | 49 | |
| IV | 4 | 0 | |
| X | 24 | 2 | |
| **Grade** | | | |
| G1 | 55 | | |
| G2 | 175 | | |
| G3 | 118 | | |
| G4 | 12 | | |
| GX | 5 | | |
| **Gender** | | | |
| Male | 119 | 191 | 153 |
| Female | 246 | 30 | 50 |
| **Age** | | | |
| $\leq 60$ | 173 | 181 | 43 |
| $>60$ | 192 | 40 | 160 |
