# Supplementary material for: Development of Prognostic Features of Hepatocellular Carcinoma Based on Metabolic Gene Classification and Immune and Oxidative Stress Characteristic Analysis
Source: Oxid Med Cell Longev. 2023 Feb 18;2023:1847700. doi: 10.1155/2023/1847700 (PMC9969974; doi:10.1155/2023/1847700)
Supplement: Supplementary 2 — Supplementary Table S2: prognosis genes in GSE14520. [file 1847700.f2.pdf]

Table S2. Prognosis genes in GSE14520 dataset

Genes	p.value	HR	Low 95%CI	High 95%CI
FH	0.0372526	0.7166647	0.523814	0.9805166
AGL	0.0023033	0.7392162	0.6086795	0.8977477
GAA	0.0054776	0.6722718	0.5079855	0.8896895
GCK	0.0409042	0.6080659	0.3774283	0.9796407
HK2	0.000237	1.5710863	1.234778	1.9989927
HK1	0.0281762	0.7057886	0.5170614	0.9634013
NNT	0.0252699	0.8109973	0.6750176	0.9743696
POR	0.0196865	0.7831195	0.6376826	0.9617263
RHD	0.0460233	0.3545794	0.1280486	0.9818662
SCD	0.0026011	1.2380389	1.0773999	1.422629
TKT	0.0410406	1.2089482	1.0077528	1.4503118
TPR	0.007821	1.5805792	1.1279927	2.2147579
UBB	0.0017776	0.3530431	0.1837541	0.6782948
PFKFB1	0.0075522	0.7682721	0.6331713	0.9321996
PFKFB3	0.0032731	1.2446927	1.0757389	1.4401822
ALDH9A1	0.0187712	0.6841382	0.4984848	0.9389357
ALDH7A1	0.0012566	0.7196185	0.589219	0.8788766
ALDH1A1	0.0185142	0.8297822	0.7104383	0.9691742
ALDH1B1	0.0149515	0.6629755	0.476138	0.9231284
SULT4A1	0.0004808	0.3216536	0.1701532	0.6080464
SULT2A1	0.0020983	0.8551374	0.7739803	0.9448043
SULT1A1	0.0028146	0.7425844	0.6108608	0.9027125
SULT1A2	0.0069443	0.7309549	0.5821955	0.9177245
SULT1B1	0.0234459	0.6300244	0.4224929	0.9394968
SLC25A10	0.005997	0.596734	0.4129117	0.8623912
GLYAT	0.0036085	0.8291239	0.7308306	0.940637
GRHPR	0.0002038	0.6734449	0.5466381	0.8296678
GSTK1	0.0017233	0.667746	0.5187187	0.8595888
GSTA1	0.0255028	0.8170764	0.6843486	0.9755464
B3GNT2	0.023944	0.5935323	0.3773936	0.933457
HPSE2	0.028858	0.3448681	0.1327406	0.8959887
SLC16A3	5.90E-05	2.685953	1.658533	4.349834
SLC35D1	0.0381127	0.7680662	0.5985215	0.9856383
MAN2C1	0.0126595	0.5986226	0.3999156	0.8960617
B4GALT5	0.0431245	1.3638781	1.009642	1.8423989
ACY1	0.0228552	0.8036161	0.6656966	0.9701099
ADH5	0.0015125	0.5661567	0.398374	0.8046042
ADH6	0.0019769	0.7817936	0.6689011	0.9137395
AOC3	0.0412636	1.4282885	1.0142324	2.0113813
BPHL	0.009605	0.7198275	0.5612753	0.9231683
CES2	0.0043373	0.8324845	0.7339506	0.9442468
CES1	0.0049735	0.8509172	0.76026	0.9523848

CYP2A13	0.0210713	0.5767926	0.3613786	0.9206126
CPT2	0.005016	0.65859	0.4919486	0.881679
CYP26B1	0.0040474	1.3017556	1.0875183	1.558197
CYP27A1	0.0067785	0.7945799	0.672741	0.9384847
CYP21A2	0.0374072	1.3107432	1.0158992	1.6911597
DCXR	0.0004747	0.7652024	0.6585552	0.8891201
DERA	0.0136682	0.7416354	0.5848075	0.94052
CYP3A43	0.0005406	0.6616345	0.5235991	0.8360599
ENO1	0.0162626	1.3123844	1.0513937	1.6381616
ENO2	0.0047319	1.3475779	1.0956254	1.6574701
CYP4F12	0.0307656	0.7592174	0.5913113	0.9748015
CYP4A11	0.0002582	0.7798404	0.6824622	0.8911133
FBP1	0.003039	0.8081134	0.7019124	0.9303829
FDX1	0.0056544	0.6921609	0.5333684	0.8982285
FMO3	0.0384553	0.9066205	0.826254	0.9948038
G6PD	0.0005562	1.313193	1.1249856	1.5328871
G6PC	0.0037064	0.8456285	0.7550923	0.94702
GCDH	0.0037029	0.7071059	0.5595557	0.8935639
GCLC	0.020313	0.753601	0.5934335	0.9569975
GOT2	0.0005496	0.6693945	0.5330948	0.840543
GYS2	0.0274195	0.8814905	0.7880141	0.9860553
HADH	0.0065662	0.6989889	0.5399087	0.904941
HAGH	5.35E-05	0.6669448	0.5479511	0.8117793
LDHA	0.0010256	2.0089719	1.3247058	3.0466902
MAOB	0.0034052	0.7494792	0.6179335	0.9090284
MAOA	0.0041864	0.7621579	0.6328728	0.9178537
MDH2	0.0043813	0.5899082	0.4103115	0.848116
NAT1	0.0202609	0.7307975	0.5607955	0.9523345
NAT2	0.0208862	0.8338719	0.7147524	0.9728438
OGDH	0.0220521	0.7135546	0.5345022	0.9525876
PCK1	0.0063076	0.8837361	0.8087322	0.9656961
PCK2	0.0016874	0.7705902	0.654919	0.9066911
PDK2	0.0030834	0.4609639	0.2760013	0.7698795
PECR	0.005511	0.7579719	0.6232604	0.9218
PHKB	0.045081	0.7245099	0.5286363	0.9929596
PYGL	0.0004192	0.6845389	0.5545576	0.8449861
RBKS	0.0343271	0.7360117	0.554112	0.9776239
SDC1	0.0422443	0.8220547	0.680428	0.9931602
SDHD	0.0074169	0.6888857	0.5244085	0.90495
SDHA	0.0344568	0.6537214	0.4408514	0.9693781
SMOX	0.0422647	1.890999	1.0225038	3.4971775
SORD	0.0026415	0.7840535	0.6690704	0.9187969
TPMT	0.0167195	0.6026625	0.3980452	0.9124644
UGP2	0.000888	0.6851044	0.5481591	0.8562625

AKR7A2	0.0259421	0.7178151	0.5361606	0.9610153
AKR7A3	0.0194217	0.8450918	0.7338536	0.9731916
AADAC	0.0161736	0.8491429	0.7431951	0.9701944
ACAT1	0.0017366	0.7153564	0.5800732	0.88219
ACADM	0.0002281	0.6349163	0.4986608	0.8084026
ACAA2	0.0019872	0.5769798	0.4071661	0.8176162
ACOX1	0.0018799	0.4746077	0.296663	0.7592873
ACSM1	0.0098443	0.7968052	0.6705762	0.9467955
ACSL6	0.0214745	0.5082052	0.285422	0.9048796
ACYP1	0.0155059	1.5464028	1.0864542	2.2010699
ADH1B	8.74E-05	0.8399858	0.7699089	0.9164411
ADH1A	0.031157	0.8921691	0.8042302	0.9897237
ALDOA	0.0046023	1.3831803	1.1051868	1.7310989
ALDH2	0.0099067	0.7501211	0.6028892	0.9333087
BPNT1	0.0427143	2.1393797	1.0252819	4.4640853
UGT1A10	0.0235062	0.3417442	0.1349644	0.8653322
UGT2B15	0.0006293	0.7204453	0.5969827	0.8694415
CHSY1	0.0072113	1.4214514	1.0998223	1.8371369
CNDP2	0.0403005	0.728707	0.5384986	0.9861008
CRYL1	0.0004028	0.728022	0.6106346	0.8679759
EHHADH	0.0001411	0.7333528	0.6251128	0.8603349
CSPG4	0.0318028	2.2857655	1.0746295	4.8618839
CYB5R3	0.0021337	2.010802	1.2875118	3.1404177
MAT2B	0.0371147	0.7381521	0.5548418	0.982025
MGST3	0.0201073	0.7101488	0.5321107	0.9477563
CYP2C8	0.0019794	0.7760136	0.6608288	0.9112754
CYP2A6	0.0073426	0.8684976	0.7834354	0.9627954
CYP7A1	0.0012563	0.8545162	0.7766652	0.9401708
CYP4F3	0.0078471	0.817022	0.7039318	0.9482808
CYP3A4	0.0047268	0.8548886	0.7667791	0.9531227
CYP3A5	0.0135809	0.8435787	0.7369908	0.965582
CYP2J2	0.0120756	0.812082	0.6902522	0.9554148
CYP2R1	0.0197838	2.4701754	1.1545975	5.2847563
ECHS1	0.0035634	0.6240656	0.4544874	0.8569168
NUP107	0.0233929	1.3718936	1.0437489	1.803204
NUP214	0.0273829	1.6450762	1.0570919	2.5601141
NUP210	0.044039	1.4979054	1.0108447	2.2196489
SUCLG2	0.0333711	0.7809065	0.621813	0.9807046
SUCLG1	0.0337499	0.6779759	0.4735807	0.9705873
SLC2A1	0.028275	1.4949387	1.0437299	2.1412071
EPHX1	0.0017261	0.8173136	0.7204317	0.927224
CHST11	0.0167547	1.5331605	1.0802475	2.1759654
NUP37	0.0163826	1.4197131	1.0664001	1.8900834
IMPAD1	0.0125399	1.357196	1.0678496	1.7249442

PPP1R3C	0.0361281	1.1950069	1.011588	1.4116829
FADS2	0.0127197	1.2589345	1.0503531	1.5089364
FADS1	0.0050785	1.2304017	1.0642912	1.4224381
