# Supplementary material for: Development of Prognostic Features of Hepatocellular Carcinoma Based on Metabolic Gene Classification and Immune and Oxidative Stress Characteristic Analysis
Source: Oxid Med Cell Longev. 2023 Feb 18;2023:1847700. doi: 10.1155/2023/1847700 (PMC9969974; doi:10.1155/2023/1847700)
Supplement: Supplementary 3 — Supplementary Table S3: prognosis genes in HCCDB18. [file 1847700.f3.pdf]

Table S3. Prognosis genes in HCCDB18 dataset

Genes	p.value	HR	Low 95%CI	High 95%CI
DERA	0.000876	3.604025	1.69366	7.669186
IDH3A	0.031844	1.925783	1.058563	3.503465
B4GALT4	6.08E-05	2.494113	1.59552	3.898793
FADS1	0.005614	1.382982	1.099414	1.739689
B3GAT3	0.006681	1.826779	1.181927	2.823456
CYP2C8	3.71E-05	0.75861	0.665289	0.865021
CYP4V2	0.004527	0.579407	0.397511	0.844538
PPP2R5D	0.002851	2.25029	1.320778	3.833958
MDH2	0.021519	1.795032	1.090046	2.955965
NUP93	0.026024	1.919285	1.080992	3.407662
NUP155	0.006939	1.964607	1.203272	3.207654
ECHS1	0.044564	0.636913	0.410126	0.989105
ACSL3	0.011832	1.656562	1.118205	2.45411
SMOX	0.048743	1.345669	1.001638	1.807863
CS	0.040437	1.680655	1.022879	2.761421
ENO1	2.13E-05	1.932679	1.42634	2.618766
PTGES3	3.50E-05	4.703781	2.259323	9.793003
SHPK	0.008244	0.377403	0.183167	0.777613
PFKFB4	0.031438	1.573351	1.041167	2.377556
ACSM2A	0.001753	0.762126	0.642884	0.903485
FDX1	0.006164	0.463698	0.267545	0.803663
NUP85	0.021306	2.042203	1.112075	3.750279
GLYATL1	0.000298	0.71428	0.595235	0.857134
GRHPR	0.015388	0.635698	0.440664	0.917052
LUM	0.032804	0.853688	0.73827	0.98715
ADH1A	0.014495	0.836713	0.725281	0.965266
ST3GAL4	0.04656	1.465735	1.005841	2.135905
NAT2	0.002284	0.664594	0.511152	0.864099
ACSM5	0.008468	0.749854	0.605209	0.929069
AKR7A2	0.018953	1.925124	1.113919	3.327085
GAPDH	7.55E-07	2.736576	1.836431	4.077935
SLC37A4	0.01141	0.617306	0.424811	0.897027
SDC2	0.019711	1.66375	1.084567	2.552229
HSPG2	0.009599	0.59877	0.406163	0.882714
TKT	0.000578	1.657615	1.243044	2.210452
NUP107	0.006123	1.856323	1.192783	2.888989
ACY3	0.038905	0.742288	0.559422	0.98493
PDHB	0.012479	2.600263	1.228653	5.503074
NUP205	0.03528	2.047416	1.050655	3.989809
HEXB	0.03066	1.744026	1.053218	2.887936
ADH1B	0.003209	0.819608	0.718038	0.935546
SEC13	0.001262	2.958855	1.530236	5.721224

CYB5B	0.038609	1.519384	1.022158	2.258482
HAGH	0.044454	0.681426	0.468778	0.990537
ACADVL	0.012398	0.591774	0.392259	0.892769
FMO2	0.003477	0.286675	0.124006	0.662729
AKR7L	0.018119	0.731961	0.565064	0.948154
HADH	0.019572	0.586596	0.374836	0.917988
UGP2	0.002785	0.515499	0.333907	0.795848
GGT5	0.003093	0.629957	0.463822	0.855599
PPP2R1A	0.018764	1.999839	1.121958	3.56462
GLO1	0.001853	2.076712	1.310807	3.290135
HK1	0.034559	0.641831	0.425432	0.968302
LDHA	0.001459	2.174992	1.347856	3.509713
GLYAT	0.000241	0.759438	0.655686	0.879608
RPE	0.005343	2.970572	1.38086	6.390438
NUP43	0.030709	2.068634	1.069903	3.99966
ADH4	0.040869	0.885128	0.787431	0.994947
CYP3A4	0.000347	0.80752	0.71826	0.907873
CYP2W1	0.001901	3.222629	1.539742	6.744855
B3GALT6	0.004566	2.043894	1.24713	3.349692
DLAT	0.008943	1.73673	1.148146	2.627044
HADHA	0.016976	2.351034	1.165329	4.743175
G6PD	0.000811	1.450914	1.166925	1.804017
RPIA	0.000353	2.761199	1.581783	4.820018
FBP1	0.033033	0.794941	0.643722	0.981683
CYP2C18	0.027085	0.765659	0.604233	0.970211
PCK2	0.018578	0.673135	0.484141	0.935907
ALDH9A1	0.001099	0.37202	0.205438	0.673677
PDP1	0.03153	0.570348	0.341874	0.951509
CHST5	0.000325	2.529707	1.525122	4.196004
PGK1	0.034297	1.55332	1.033121	2.33545
SLC35B2	0.001856	2.160278	1.330017	3.508827
HS2ST1	0.009353	2.469479	1.248879	4.883041
ACSL6	0.031954	0.66487	0.457893	0.965406
ACACA	0.003501	2.270553	1.309442	3.937107
SLC26A2	0.021778	1.940375	1.101394	3.418447
ACOT7	0.000759	2.047924	1.349297	3.108281
CHST7	0.001911	0.358834	0.18785	0.685451
CYP4A11	0.014236	0.812342	0.687969	0.959201
GGT1	0.049251	1.276731	1.000802	1.628735
SLC16A1	0.005267	1.530277	1.134959	2.063288
CYP4F2	0.002731	0.749913	0.621237	0.905242
CYP21A2	0.01736	0.653347	0.460096	0.927768
AHCY	5.00E-08	3.405148	2.191835	5.290102
MAOB	0.048855	0.743613	0.553784	0.998514

UGT2B15	0.000482	0.772779	0.668656	0.893116
SLC35D2	0.000807	2.612206	1.489557	4.580973
IDS	0.005826	0.518246	0.324811	0.826879
MAT2A	0.010472	2.115674	1.191971	3.755191
CPT1B	0.005634	0.64915	0.478066	0.881461
HYAL3	0.004052	1.801576	1.205903	2.691492
POM121C	0.009496	2.778674	1.283636	6.014965
ELOVL5	0.010694	1.982175	1.172144	3.351991
TRMT112	0.026284	1.954292	1.082218	3.529102
CYP2C19	0.003182	0.628411	0.461508	0.855674
SEH1L	1.70E-05	3.22539	1.891626	5.499575
GYG1	0.042088	1.510004	1.014845	2.246757
CHST13	0.000495	1.884271	1.319277	2.69123
SUCLG1	0.022822	1.955561	1.097715	3.483801
POM121	0.03622	2.069699	1.047813	4.088186
VDAC1	0.003302	2.482511	1.353533	4.553166
OMD	0.029122	0.332024	0.123319	0.89394
ACACB	0.019654	0.585099	0.372964	0.91789
CYP27A1	0.022808	0.700382	0.515446	0.95167
FUT2	0.036755	1.545623	1.027155	2.325793
B4GALT2	0.010103	1.802937	1.150629	2.825048
GPC5	0.007769	1.693737	1.149074	2.496572
UGT2B28	0.03095	2.008195	1.065986	3.783212
ACYP2	0.010197	0.459457	0.253845	0.83161
AADAC	0.024683	0.790071	0.643248	0.970408
ACSM2B	0.000738	0.732023	0.610737	0.877395
LDHAL6B	0.021005	0.006668	9.46E-05	0.469839
PGD	0.000947	1.720721	1.247265	2.373899
B3GAT1	0.019426	0.275308	0.093339	0.812036
ACADL	4.29E-05	0.562025	0.426456	0.74069
DCN	0.013638	0.836052	0.725172	0.963886
CES2	0.0116	0.754608	0.606416	0.939014
HSP90AB1	0.012738	1.735291	1.124687	2.677397
GGCT	3.77E-06	3.195992	1.953053	5.229949
ACAT2	0.005346	1.758996	1.182183	2.61725
CYP4A22	0.038898	0.799551	0.646616	0.988659
PFKFB1	0.005952	0.725909	0.577759	0.912048
GCKR	0.029338	0.78228	0.627249	0.975629
CES3	0.00667	0.69684	0.536788	0.904614
PRELP	0.019912	0.664495	0.471027	0.937427
PGP	0.005619	2.043267	1.232218	3.388151
FMO3	4.08E-05	0.698857	0.588916	0.829322
CYP3A5	6.88E-05	0.711259	0.6014	0.841186
TPMT	0.000358	0.256868	0.121781	0.5418

IDH3B	0.010372	2.447546	1.23448	4.852634
HMMR	2.58E-05	2.498892	1.631063	3.828461
GPC3	0.009526	1.218061	1.049311	1.413949
MTRR	0.048495	2.093142	1.004895	4.359903
PGAM1	0.01932	1.74607	1.09459	2.785299
ALDOA	0.016078	1.434395	1.069312	1.924123
ACAT1	0.004988	0.61724	0.440739	0.864425
AKR7A3	0.016216	0.806108	0.676203	0.96097
CYP2A6	0.006455	0.850153	0.756422	0.955498
ALDOB	0.026975	0.863941	0.758931	0.983481
HS3ST2	0.045492	0.582386	0.342871	0.989217
ADH1C	2.48E-07	0.752422	0.675348	0.838293
SULT1B1	0.01675	0.582882	0.374553	0.907084
ALDH2	0.022862	0.67831	0.485564	0.947567
MDH1	0.025543	1.915894	1.082769	3.390056
DLD	0.045634	1.692837	1.01027	2.836565
EHHADH	0.024492	0.756315	0.592938	0.964709
PHKG1	0.033026	0.148849	0.025832	0.857687
DSE	0.009543	0.481231	0.276797	0.836656
D2HGDH	0.003582	0.48611	0.299187	0.789817
ACSM1	0.005947	0.779388	0.652565	0.930857
NUP37	0.003151	2.210499	1.30562	3.742516
NUP35	0.000456	3.423778	1.720425	6.813584
TPI1	5.87E-07	3.026742	1.960039	4.673972
CYP2C9	0.009757	0.831318	0.722634	0.956348
CYP4F3	0.043283	0.778366	0.610453	0.992465
IDUA	0.027378	0.565392	0.340666	0.938362
SUCLA2	0.009673	2.684721	1.270557	5.672885
B3GNT3	0.016793	1.444205	1.068535	1.951951
GSTM2	0.001729	0.516228	0.341358	0.78068
SLC2A1	0.025969	1.365704	1.038038	1.7968
GYS2	0.005092	0.754129	0.619005	0.918749
RPS27A	2.16E-05	2.792891	1.738754	4.48611
NAT1	0.003252	0.397474	0.215003	0.734808
CYP8B1	0.006644	0.826448	0.720171	0.94841
CYP1A2	0.009939	0.721232	0.562565	0.924651
HS6ST3	0.028975	38.95298	1.455609	1042.406
FUT9	0.000138	81.73271	8.494185	786.4481
PRKACG	0.026238	9.21E+25	1171.707	7.23E+48
