# Supplementary material for: Development of Prognostic Features of Hepatocellular Carcinoma Based on Metabolic Gene Classification and Immune and Oxidative Stress Characteristic Analysis
Source: Oxid Med Cell Longev. 2023 Feb 18;2023:1847700. doi: 10.1155/2023/1847700 (PMC9969974; doi:10.1155/2023/1847700)
Supplement: Supplementary 4 — Supplementary Table S4: prognosis genes in TCGA. [file 1847700.f4.pdf]

Table S4. Prognosis genes in TCGA dataset

Genes	p.value	HR	Low 95%CI	High 95%CI
SLC2A1	6.23E-08	1.404285	1.241808	1.58802
LDHA	4.73E-07	1.797589	1.430826	2.258364
ALDOA	1.45E-05	1.36001	1.183541	1.562791
PRKACB	0.009016	1.348389	1.077419	1.687507
CSPG4	0.044247	1.21524	1.00503	1.469417
XYLT2	0.02354	1.355974	1.04181	1.764877
MANBA	0.001817	1.484082	1.157981	1.902016
CYP8B1	0.015662	0.934055	0.88378	0.98719
BPGM	0.033611	1.333604	1.022573	1.73924
ENO1	5.52E-08	1.605688	1.353558	1.904781
NUP210	0.000937	1.356493	1.132356	1.624996
GLYAT	0.037058	0.930304	0.869227	0.995673
MAT1A	0.037672	0.907753	0.828573	0.9945
TRMT112	0.001041	1.683545	1.233141	2.298459
PCK1	0.02286	0.9264	0.86737	0.989448
GNPDA1	1.64E-06	1.642391	1.340811	2.011803
FBP1	0.017403	0.895235	0.817192	0.98073
ADH4	1.35E-05	0.893516	0.849335	0.939994
NUP58	0.009495	1.396161	1.08496	1.796626
SDC3	0.003792	1.300278	1.088505	1.553253
NUP98	0.01529	1.407877	1.067854	1.856168
ACSM2A	0.004296	0.880634	0.807058	0.960917
NUP85	3.02E-06	1.825171	1.417814	2.349566
RPIA	3.87E-05	1.703765	1.321886	2.195966
ADH1A	0.006141	0.904956	0.842567	0.971965
BPNT1	0.029051	1.347272	1.030898	1.76074
CYP11A1	0.017749	0.861593	0.761756	0.974514
FDX2	0.005335	1.366319	1.096967	1.701809
MTRR	0.009493	1.380632	1.082003	1.761681
AKR7A3	0.005742	0.894689	0.82676	0.968199
B3GALT6	0.027598	1.367672	1.035141	1.807028
NUP188	0.000583	1.49073	1.187384	1.871572
HSP90AB1	0.002343	1.414431	1.131334	1.768369
CYP3A4	0.022136	0.946566	0.903069	0.992157
GCDH	0.007787	0.761161	0.622563	0.930613
FMO2	0.027272	0.725997	0.546347	0.964718
SGSH	0.011775	1.41513	1.080084	1.854109
ACAT1	0.000743	0.740303	0.621628	0.881634
CS	0.03582	1.247816	1.014769	1.534384
FMO3	0.003538	0.897945	0.835284	0.965307
ALDH2	0.000258	0.725487	0.610776	0.861743
PAPSS1	0.001136	1.384102	1.138017	1.6834

HYAL3	0.04711	1.178032	1.002094	1.384859
BAAT	0.018158	0.894079	0.814769	0.981109
B4GALT5	0.000218	1.390953	1.1677	1.656891
GYS2	0.00293	0.889149	0.822922	0.960707
TBXAS1	0.038865	1.248845	1.01143	1.541988
HS2ST1	0.000364	1.582034	1.229387	2.035837
SULT1C2	0.007407	1.157915	1.040094	1.289083
ST3GAL4	0.004273	1.33562	1.095141	1.628905
FUT11	0.011633	1.39293	1.07677	1.801921
ADH1B	0.001709	0.896665	0.837587	0.95991
UBC	0.012758	1.514766	1.092484	2.100275
PPARD	0.014249	1.2706	1.049144	1.538802
FUT10	0.011009	1.509553	1.098936	2.073597
CRYL1	0.046675	0.847677	0.720306	0.997571
GPC1	0.000564	1.265453	1.106955	1.446646
PGK1	0.000831	1.375429	1.140929	1.658126
NDC1	3.03E-07	1.828368	1.451344	2.303335
SLC9A1	0.031912	1.217466	1.017163	1.457212
B4GALT6	0.004879	1.388808	1.104904	1.74566
PKM	0.000157	1.203767	1.093408	1.325266
CYP2C9	9.39E-05	0.87919	0.824182	0.93787
NUP54	0.005296	1.477343	1.12294	1.943597
PGM2L1	0.00871	1.353156	1.079469	1.696232
NUP35	0.007809	1.503427	1.113304	2.030257
HAGH	0.001774	0.740585	0.613465	0.894046
GALNS	0.000139	1.528756	1.228917	1.901752
ACOT7	0.000103	1.531918	1.235201	1.899912
LDHD	0.000336	0.785102	0.687843	0.896112
SLC37A1	0.017948	1.274635	1.042585	1.558334
GGT7	0.006605	1.34796	1.08668	1.672062
PPP2CA	0.000883	1.937987	1.312152	2.862316
G6PC3	0.001368	1.347388	1.122568	1.617233
ALDOB	0.00996	0.921056	0.865218	0.980497
GSS	0.00059	1.72754	1.264738	2.359695
GAPDH	2.16E-05	1.577504	1.278278	1.946774
GLO1	0.029642	1.301631	1.026388	1.650685
TPR	0.014708	1.288311	1.051057	1.57912
GYS1	0.004283	1.370005	1.103859	1.700321
CHST12	0.002542	1.64604	1.190952	2.275028
PYGB	0.002032	1.295769	1.099129	1.527589
PHKA2	6.96E-05	1.64558	1.287454	2.103324
B4GAT1	0.049949	1.256409	1.000051	1.578483
NUP205	2.27E-05	1.604697	1.289378	1.997128
AGRN	0.000339	1.290797	1.122593	1.484205

EXT2	0.003404	1.527973	1.150531	2.02924
B3GNT7	0.007092	1.191604	1.048849	1.353789
ABCC5	2.21E-06	1.878854	1.447023	2.439554
NDST2	0.002993	2.59188	1.382052	4.860774
B4GALT2	9.16E-06	1.79504	1.386179	2.324496
MAT2A	0.021112	1.220549	1.030356	1.445849
ECHS1	0.008402	0.727818	0.57466	0.921795
CYP2S1	0.019481	1.168594	1.025409	1.331774
AIP	0.019829	1.406387	1.055596	1.87375
IDH3G	0.03291	1.313874	1.022414	1.688422
SORD	0.006817	0.851114	0.757306	0.956543
CYB5B	0.036098	1.300976	1.017202	1.663914
CHST11	0.007533	1.211894	1.052564	1.395342
CYB5R3	0.000616	1.546301	1.204923	1.984399
SLC37A2	0.003798	1.353959	1.102781	1.662349
GLYATL1	0.001135	0.858253	0.782777	0.941006
CYP3A43	0.006308	0.803447	0.686681	0.940068
CYP2R1	0.04176	1.357308	1.011461	1.82141
GCLM	0.00012	1.404908	1.181479	1.67059
PTGS1	0.001331	1.29448	1.105692	1.515501
HACD2	0.018352	1.22836	1.035362	1.457334
PFKFB4	3.14E-08	1.598565	1.353836	1.887533
ACADS	0.006198	0.738897	0.594951	0.917669
CHST1	0.002332	1.26062	1.08599	1.463331
CHPF2	0.00031	1.608194	1.242224	2.081982
HMMR	8.41E-07	1.449992	1.250707	1.681031
NUP160	0.005414	1.380683	1.099943	1.733075
AS3MT	0.009578	0.769636	0.63133	0.938241
GALE	0.024439	1.327244	1.037185	1.69842
AHCY	0.030733	1.296574	1.024402	1.641058
NUP155	6.40E-06	1.728065	1.362642	2.191485
TPI1	6.05E-05	1.650077	1.291862	2.10762
HPSE	0.029005	1.277946	1.025417	1.592664
B4GALT7	0.013135	1.454993	1.081819	1.956893
SLC35B2	0.006272	1.36147	1.091218	1.698655
SLC25A12	0.037702	1.237528	1.012187	1.513037
UGP2	0.023009	0.798542	0.657751	0.96947
YOD1	0.016017	1.33075	1.054658	1.679118
CALM1	0.002817	1.633778	1.183877	2.254652
RPE	8.72E-05	1.751125	1.323657	2.316642
PGD	5.40E-06	1.513676	1.266074	1.8097
B4GALT4	0.000668	1.558863	1.207101	2.013131
EHHADH	0.021571	0.878558	0.7867	0.981142
GLB1L	0.029426	1.218487	1.019957	1.455659

UGDH	0.001633	1.28272	1.098622	1.497667
AAAS	0.002452	1.713281	1.209305	2.42729
GNS	0.005583	1.363871	1.095109	1.698593
SULT2A1	0.01188	0.922231	0.865858	0.982274
ACSL6	0.027343	0.826922	0.698479	0.978984
ENO2	0.002891	1.198014	1.063756	1.349218
PDK4	0.014201	0.880679	0.79563	0.97482
NUP214	0.024516	1.433192	1.047333	1.96121
NUP43	4.42E-06	1.853027	1.423987	2.411334
GNPDA2	0.001798	1.571103	1.183091	2.08637
BSG	0.000149	1.420415	1.184853	1.702809
ACACA	0.000303	1.5019	1.204524	1.872692
CYP4V2	0.015253	0.808618	0.681113	0.959993
G6PD	1.07E-09	1.386029	1.247981	1.539347
PPP2R5D	0.012416	1.33964	1.065208	1.684774
HYAL2	0.024744	1.389064	1.042656	1.85056
HSD17B12	0.00077	1.523345	1.192	1.946797
UGT1A6	0.021191	1.129301	1.018349	1.252342
CYP27A1	0.004366	0.850204	0.760438	0.950568
GGCT	0.000447	1.656565	1.249772	2.195768
PFKFB3	0.00459	1.161731	1.047339	1.288616
DLAT	3.12E-05	1.580616	1.274235	1.960664
ECI1	0.006077	0.670571	0.504035	0.892132
VCAN	0.032582	1.121894	1.009584	1.246697
CYP3A5	0.007051	0.870995	0.787738	0.963051
ME1	0.000674	1.199506	1.080085	1.332131
HADHA	0.000428	1.87096	1.320264	2.651355
GYG1	4.36E-05	1.653096	1.299027	2.103672
AKR1B1	0.031059	1.142373	1.012194	1.289293
TALDO1	5.57E-05	1.522993	1.241228	1.86872
NUP107	4.24E-05	1.651	1.298689	2.098887
HEXB	0.000348	1.687302	1.266733	2.247503
ACYP1	3.49E-07	1.870727	1.470191	2.380386
PDK3	0.000286	1.458755	1.189548	1.788886
CYP7A1	0.004153	0.908505	0.850808	0.970114
SLC26A2	0.001803	1.407934	1.135723	1.745389
ACSL3	0.000875	1.43337	1.159493	1.771939
VDAC1	0.001266	1.646785	1.215951	2.23027
ACSS1	0.020612	1.182507	1.026044	1.362829
PPP2R1A	0.007245	1.392418	1.093546	1.772973
ST3GAL2	0.003037	1.605809	1.174021	2.196403
GRHPR	0.023027	0.802407	0.663675	0.970139
NUP93	3.26E-05	1.699621	1.323344	2.182888
SEH1L	0.000389	1.823848	1.308558	2.542052

PTGES3	0.000369	1.775481	1.294498	2.435178
ADHFE1	0.018259	0.847211	0.73824	0.972267
ACADL	0.019767	0.869593	0.773189	0.978017
2-Mar	0.000206	0.749007	0.643001	0.87249
SLC16A3	7.43E-07	1.326289	1.185977	1.483201
PGP	0.014335	1.272891	1.049342	1.544065
GSTM3	0.031925	1.146988	1.011921	1.300083
NUP50	0.008858	1.411477	1.090431	1.827047
TKT	3.14E-05	1.350137	1.172178	1.555113
CYP2C8	0.025178	0.927575	0.868487	0.990684
CHP1	0.019804	0.749333	0.587834	0.955202
GSTZ1	0.038822	0.831984	0.698767	0.990598
B4GALT3	7.14E-05	1.676861	1.299299	2.164138
ME2	0.000469	1.491699	1.192253	1.866352
ME3	0.041836	1.211871	1.007125	1.458241
IDH3B	0.005581	1.557829	1.138599	2.131419
SMOX	5.35E-06	1.371374	1.19696	1.571202
ACOT12	0.000247	0.838062	0.762522	0.921084
GOT2	0.00012	0.667349	0.543043	0.82011
HK2	0.000743	1.21354	1.084464	1.357978
B3GAT3	3.57E-06	1.811124	1.408854	2.328254
NCOA1	0.049737	1.296173	1.000298	1.679565
UGT2B15	0.005904	0.911988	0.854092	0.973809
CHAC2	4.23E-06	1.905096	1.447601	2.507176
SEC13	0.000212	1.772886	1.309471	2.400302
RAE1	7.95E-06	1.895719	1.431801	2.509951
CYP4A11	0.045338	0.921245	0.850139	0.998299
PGAM1	0.031083	1.300288	1.024164	1.650857
ADH1C	0.002907	0.9178	0.867412	0.971115
SLC35D2	0.008338	1.404081	1.091153	1.806752
PFKP	0.000192	1.20361	1.091905	1.326742
GLB1	0.003526	1.448525	1.129343	1.857915
PECR	0.036606	0.851885	0.732989	0.990066
G6PC	0.000444	0.881634	0.821784	0.945843
NUP37	2.41E-05	1.732634	1.342486	2.236166
NUP153	0.049613	1.200148	1.000308	1.439911
NUP62	3.25E-05	1.627449	1.29344	2.04771
HK3	0.036714	1.204431	1.01155	1.434091
ACY3	0.033493	0.877532	0.777966	0.989842
RPS27A	0.008058	1.329557	1.076959	1.641401
