# Supplementary figures and images for: Development of Prognostic Features of Hepatocellular Carcinoma Based on Metabolic Gene Classification and Immune and Oxidative Stress Characteristic Analysis
Source: Oxid Med Cell Longev. 2023 Feb 18;2023:1847700. doi: 10.1155/2023/1847700 (PMC9969974; doi:10.1155/2023/1847700)

**S7\_Fig.1. There were significant differences in the pancancer immunotypes**

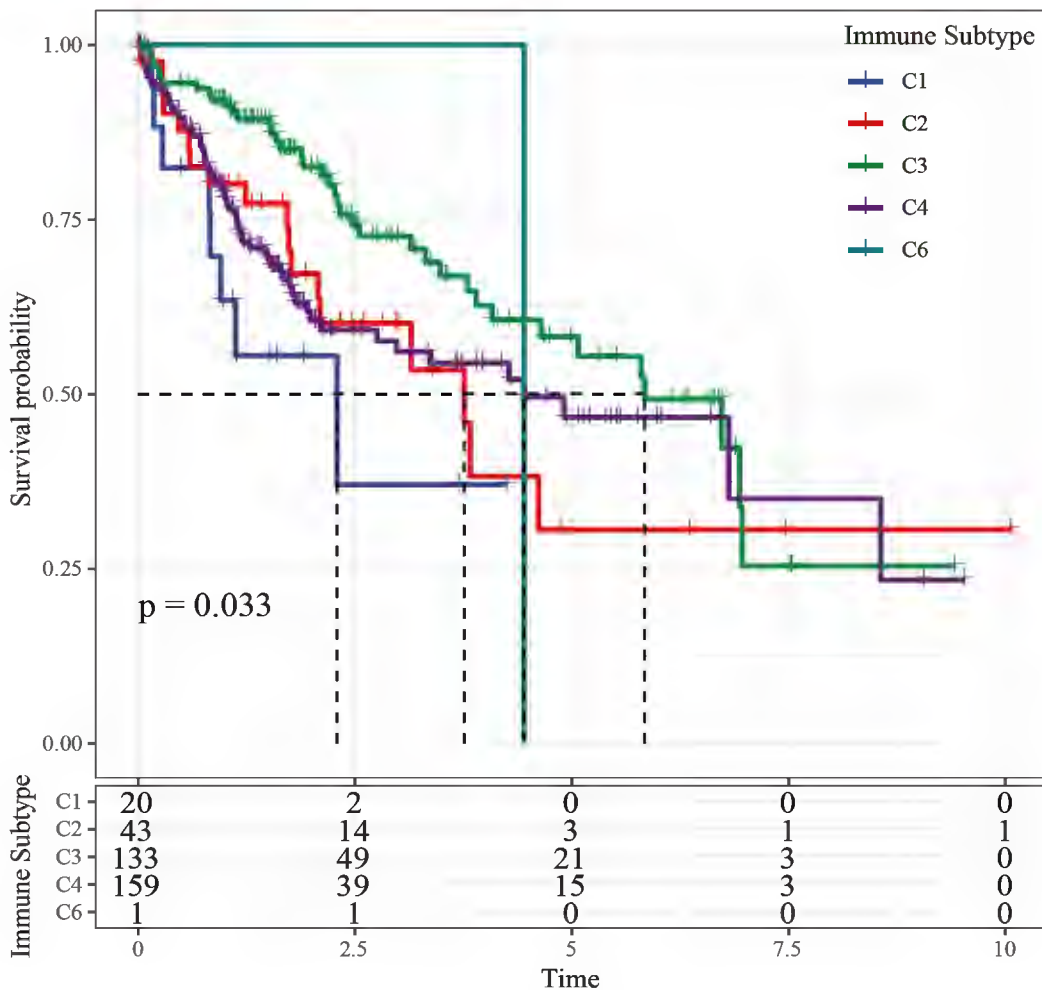

Supplement: Supplementary 5 — Supplementary Figure S1: there were remarkable variations in the pan-cancer immunotypes. [file 1847700.f5.pdf]
