# Supplementary material for: Development of Prognostic Features of Hepatocellular Carcinoma Based on Metabolic Gene Classification and Immune and Oxidative Stress Characteristic Analysis
Source: Oxid Med Cell Longev. 2023 Feb 18;2023:1847700. doi: 10.1155/2023/1847700 (PMC9969974; doi:10.1155/2023/1847700)

S8_Fig. 2. Dark module was related to tumor processes.(a)Biological process;(b)cellular component;(c)molecular function;(d)KEGG pathway

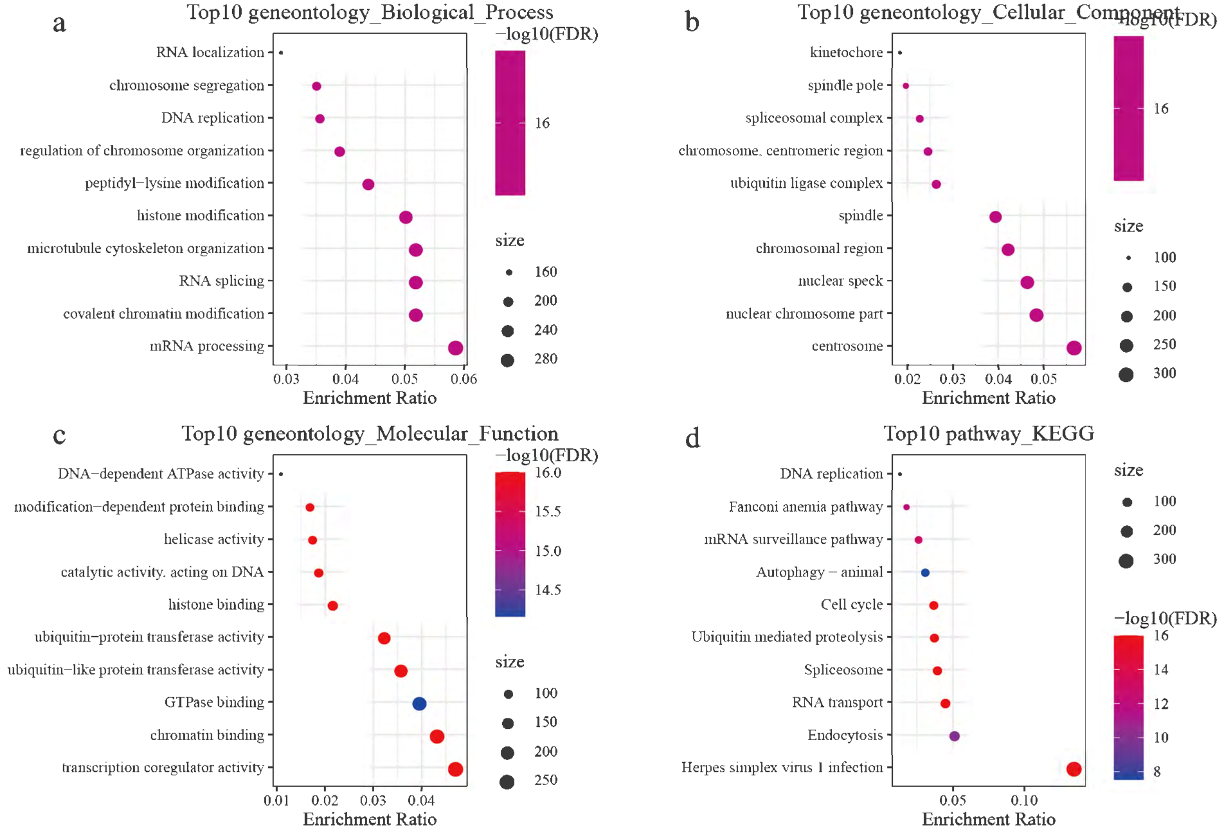

Supplement: Supplementary 6 — Supplementary Figure S2: the dark module was related to tumor processes. (a) Biological process; (b) cellular component; (c) molecular function; (d) KEGG pathway. [file 1847700.f6.pdf]
