# Supplementary material for: Development of Prognostic Features of Hepatocellular Carcinoma Based on Metabolic Gene Classification and Immune and Oxidative Stress Characteristic Analysis
Source: Oxid Med Cell Longev. 2023 Feb 18;2023:1847700. doi: 10.1155/2023/1847700 (PMC9969974; doi:10.1155/2023/1847700)

S9_Fig. 3.Green module was related to nervous system disease.(a)Biological process; (b)cellular component;(c)molecular function;(d)KEGG pathway

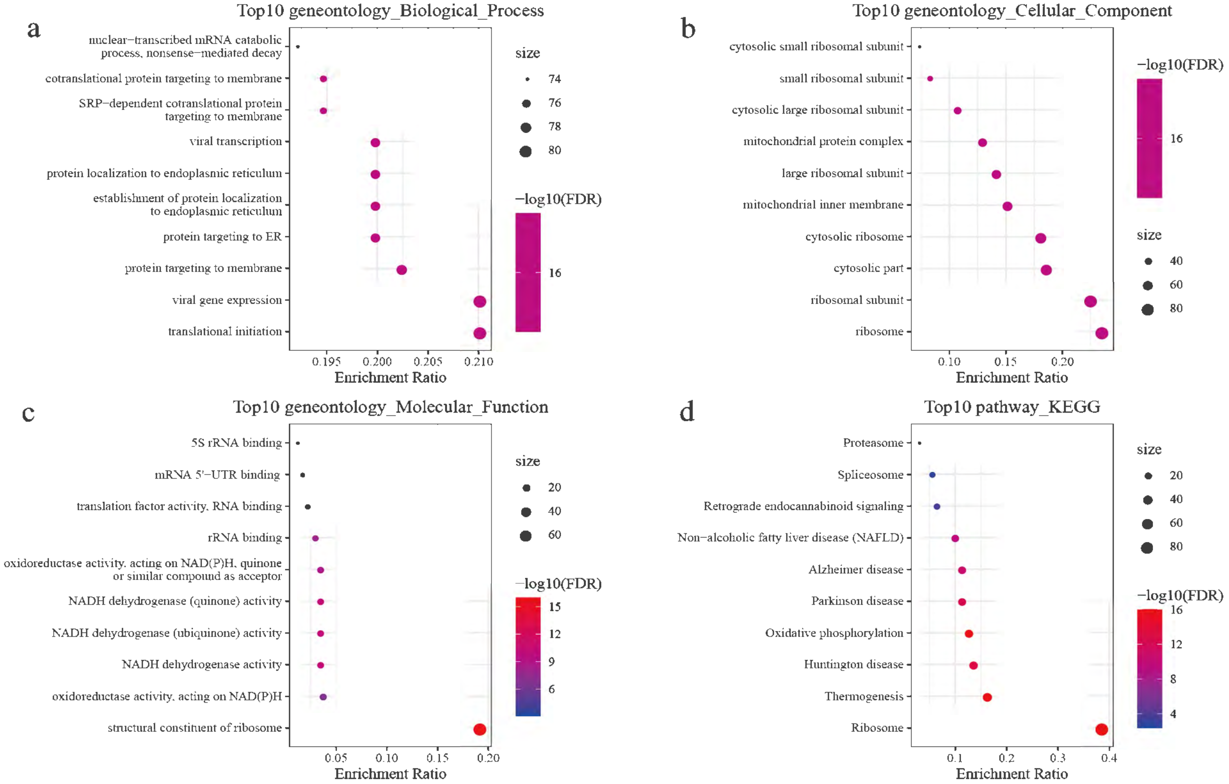

Supplement: Supplementary 7 — Supplementary Figure S3: green module was related to nervous system disease. (a) Biological process; (b) cellular component; (c) molecular function; (d) KEGG pathway. [file 1847700.f7.pdf]
